# Treatments to post-stroke depression, which is more effective to HAMD improvement? A network meta-analysis

**DOI:** 10.3389/fphar.2022.1035895

**Published:** 2022-12-19

**Authors:** Jie Zhang, Zhaoming Song, Chen Gui, Guannan Jiang, Wei Cheng, Wanchun You, Zhong Wang, Gang Chen

**Affiliations:** Department of Neurosurgery and Brain and Nerve Research Laboratory, The First Affiliated Hospital of Soochow University, Suzhou, China

**Keywords:** post-stroke depression, antidepressant, adjuvant therapy, meta-analysis, Hamilton depression scale (HAMD)

## Abstract

**Introduction:** Post-stroke depression (PSD) is a common mental health problem after cerebrovascular accidents. There are several treatments that have been shown to be effective in treating post-stroke depression. However, it is not clear which treatment is more effective.

**Methods:** In this meta-analysis, an appropriate search strategy was used to search eligible randomized controlled trials (RCTs) on different treatments to treat patients with Post-stroke depression published up to December 2021 from the CNKI, PubMed, and Cochrane Library. We assessed the mean difference or odds ratio between each treatment and placebo and summarized them as the average and 95% confidence interval (CI) by conducting Bayesian network meta-analyses.

**Results:** By constructing a Bayesian network meta-analysis, we found that acupuncture combined with fluoxetine (vs placebo MD, −8.9; 95% CI, [−15, −2.9]) or paroxetine (vs placebo MD,—8.5; 95% CI, [−15, −2.5]) was the most effective for change in Hamilton depression scale (HAMD) at the end of the 4th week. For change in Hamilton depression scale at the end of the 8th week, rTMS combined with paroxetine (vs placebo MD, −13; 95% CI, [−17, −7.9]) had the greatest amount of change. The efficacy of medication combined with adjuvant therapy was also superior for the percentage of patients with Hamilton depression scale change over 50%.

**Discussion:** The combination of antidepressants with adjuvant therapy may enhance the efficacy of antidepressants and achieve better results than antidepressant monotherapy in both Hamilton depression scale changes at the end of week 4 or 8 and 50% Hamilton depression scale improvement rate. Acupuncture combined with fluoxetine treatment was more effective in the treatment of post-stroke depression at week 4, while rTMS combined with paroxetine was more effective at week 8. Further research is needed to determine whether acupuncture combined with fluoxetine is better than rTMS combined with paroxetine for post-stroke depression at week 8.

## Background

Stroke is the third leading cause of death worldwide, accounting for approximately 5.9 million deaths each year. Stroke morbidity and mortality are rising rapidly with the growth of the elderly population (Villa et al., 2018). Cerebrovascular accidents and depression following are currently the two major causes of economic burden on society (Medeiros et al., 2020). Post-stroke depression (PSD) is the most common and burdensome neuropsychiatric complication after stroke. Some studies have shown that PSD is about one-third of the prevalence in stroke patients (Mitchell et al., 2017; Shi et al., 2017; Taylor-Rowan et al., 2019). Patients with PSD have higher mortality, more significant cognitive deficits, and lower quality of life than patients without PSD (Hilari et al., 2012; Blöchl et al., 2019; Starkstein and Hayhow, 2019). Therefore, it is extremely important to understand the pathogenesis of PSD and to find effective treatments.

The pathophysiology of PSD is complex and multifactorial, resulting from a combination of ischemia-induced neurobiological dysfunction and psychosocial distress (Robinson and Jorge, 2016). The pathology of PSD development may be related to alterations in the ascending monoamine pathway, excess of pro-inflammatory cytokines, dysfunction of the hypothalamic-pituitary-adrenal axis and altered neuroplasticity (Villa et al., 2018). The complexity of the pathogenesis of PSD makes its prevention and treatment on a biological basis a difficult task. Currently, selective 5-hydroxytryptamine reuptake inhibitors and tricyclic antidepressants have been clinically shown to have positive effects in the prevention and treatment of PSD, but the mechanisms by which they exert their therapeutic effects remain unclear and are associated with serious adverse events (Paolucci, 2017; Das, 2018). In recent years, with the continuous research on the pathology of PSD, many new effective treatment methods have emerged, such as acupuncture, herbal medicine, hyperbaric oxygen (HBO) and repetitive transcranial magnetic stimulation (rTMS), or even combined treatments (Lefaucheur et al., 2020; Liang et al., 2020; Gong et al., 2021). Treatments for PSD are longer restricted in medications alone.

There are currently a variety of treatments for PSD. There are also relevant studies describing the effectiveness of different treatments. But previous studies comparing the efficacy of these treatments are inadequate, especially there is no systematic assertion on the effectiveness of adjuvant therapy. Therefore, a Bayesian network meta-analysis was performed in order to analyze which of the treatments is more beneficial for the improvement of post-stroke depressed patients. The differences in the effectiveness of different treatments for PSD were analyzed in order to be able to provide an evidence-based medical reference for clinical treatment of PSD.

## Materials and methods

### Study protocol

Before starting this study, we prepared a draft study protocol following the format of the Cochrane Collaboration. The meta-analysis has not yet been registered.

### Literature search

In our study, we used an appropriate search method to screen eligible randomized controlled trails on treatments to patients with PSD in 3 literature databases, PubMed, China National Knowledge Infrastructure (CNKI) and Cochrane library. The publication date of the included papers was up to December 2021. The search was performed in both databases following the keywords: “post-stroke depression” AND (“citalopram” OR “amitriptyline” OR “paroxetine” OR “fluoxetine” OR “venlafaxine” OR “sertraline” OR “mirtazapine” OR “escitalopram” OR “duloxetine” OR “antidepressant” OR “shuganjieyutang” OR “hyperbaric oxygen” OR “acupuncture” OR “repetitive transcranial magnetic stimulation” OR “rTMS” OR “HBO”) AND (“randomized controlled trail” OR “RCT” OR “random” OR “controlled”).

### Inclusion and exclusion criteria

The criteria for study selection are described below. 1) Randomized controlled studies involving the treatment of post-stroke depressed patients. 2) Each included article must contain at least one outcome indicator, including Hamilton depression scale (HAMD) change at week 4, HAMD change at week 8, and HAMD 50% remission rate at end of treatment. 3) All data enrolled was assessed by HAMD-17. 4) Herbal medicine included was limited to traditional Chinese medicine, “shuganjieyutang”. 5) Each article must include at least two treatment modalities (including placebo) for each other. Studies that do not meet the 3 criteria above will be excluded.

### Quality assessment and data extraction

Following the Cochrane Collaboration’s risk analysis assessment form, 2 authors were involved in collating and summarizing the quality of each article, including authorship, year of publication, and patient-based information. The potential risk of bias for each article was identified and the quality of the article was assessed, but in case of disagreement between the 2 authors, the other author was brought in for final determination.

### Statistical analysis

After recording data from literatures that met the criteria, we used Gemtc R-package and R4.0.3 software to construct a Bayesian network model (van Valkenhoef et al., 2012; van Valkenhoef et al., 2016). We first tested the heterogeneity of the model, using chi-square q-tests and *I* ^*2*^ statistics to assess heterogeneity between trials. If *p* < 0.05 or *I* ^*2*^ > 50% showed significant heterogeneity, a random effects model was used. On the contrary, without significant heterogeneity, a fixed-effects model was used. Subsequently, we built the corresponding models based on the above results to integrate the treatment effects of drugs in different studies. The results were presented by constructing forest plots, league tables, and ranking plots. We then validated the consistency in the network model by comparing each node with the existence of direct, indirect, and pooled evidence using the node splitting method to assess the statistical differences between the different evidence determining the consistency of the indirect evidence with the direct evidence. Similarity was judged manually based on the quality control mitigation.

## Result

### Study charachteristics

**FIGURE 1:**
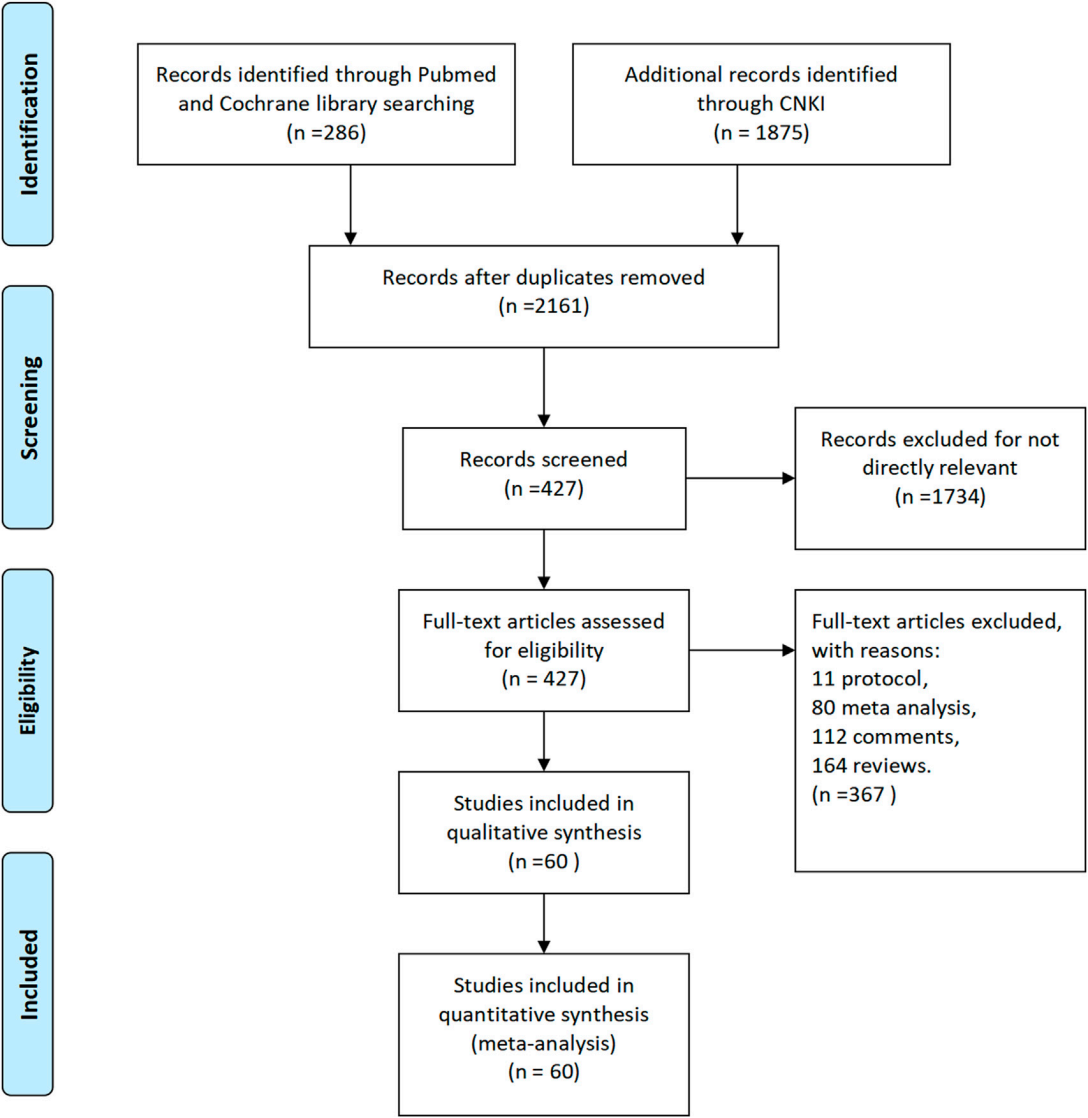
Flow diagram for study identification.

According to the above method, we searched 286 articles in Pubmed and Cochrane Library and 1875 articles in CNKI. By comparison, we removed the overlapping articles and obtained the final 427 articles by initial screening of abstracts. By reading the full text, 11 protocols, 80 meta-analyses, 112 comments and 164 reviews were finally removed (Figure 1). Finally, the characteristic information of the remaining 60 papers, which were included in this paper, is shown in supplementary material table one ^17-76^. This Table1 contains 16 different treatments include acupuncture, acupuncture combine with fluoxetine, acupuncture combine with paroxetine, amitriptyline, citalopram, duloxetine, escitalopram, fluoxetine, fluoxetine combine with HBO, HBO, mirtazapine, paroxetine, rTMS combine with paroxetine, sertraline, shuganjieyutang and venlafaxine.

**TABLE 1:** Characteristics of the included studies and outcome events.

Study	Countries	Publications	Treatment group, (no. Of participants)	Baseline of HAMD (Mean ± SD)	Female (%)	Mean age ±SD (year)	Outcome events
Fruehwald et al. (2003)	Austria	J Neurol	FLU (26) vs PLA (24)	FLU 32.8±12.7	FLU 53.8%	FLU 64.8±13.8	a
				PLA 30.3±15.0	PLA 29.1%	PLA 64.0±14.3	
Dong et al. (2003)	China	Chinese Joumal of Clinical Rehabilitation	ACU (42) vs FLU (38)	ACU 27.3±5.3	ACU 42.5%	N/A	a
				FLU 27.7±7.4	FLU 45.6%		
Zhang, (2005)	China	Journal of Traditional Chinese Medicine	ACU (45) vs FLU (45)	ACU 23.78±4.57	ACU 44.4%	ACU 55.5±6.27	a,c
				FLU 25.36±3.58	FLU 40%	FLU 58.1±8.91	
Zhao et al. (2005)	China	Chinese Journal of Clinical Rehabilitati	CIT (42) vs.VEN (40)	CIT 32.3±8.4	CIT 42.8%	CIT 58.16±8.49	a,c
				VEN 32.4±9.3	VEN 47.5%	VEN 61.45±8.24	
Gu and Chang, (2017)	Korea	Brain Stimulation	rTMS (12)	rTMS 10±1.3	rTMS 50%	rTMS 58.1±8.7	a
			vs PLA (12)	PLA 10±0.9	PLA 71.42%	PLA 58.3±7.8	
Zhao and Li, (2006)	China	journal of clinical acupuncture and moxibustion	ACU (23) vs FLU (21)	ACU 24.8±1.3	ACU 47.8%	N/A	a,c
				FLU 25.2±1.5	FLU 52.3%		
Luo et al. (2006)	China	Chin J Rehabil Theory Practice	PAR (42) vs.AMI (40)	PAR 28.8±6.7	PAR 26.1%	PAR 61.2±4.5	a,b,c
				AMI 29.2±5.1	AMI 30%	AMI 60.5±5.3	
Dong et al. (2007)	China	Chinese Acupuncture	ACU (36) vs FLU (34)	ACU 23.98±3.69	ACU 36.1%	ACU 59.21±7.56	a,c
				FLU 24.12±3.17	FLU 44.1%	FLU 56.61±8.21	
Li, (2007)	China	Modern Journal of Integrated Traditional Chinese and Western Medicine	PAR (44) vs.PLA (42)	PAR 23.9±5.78	PAR 36.3%	PAR 59.2±12	a,b
				PLA 23.7±7.54	PLA 38.1%	PLA 58.9±12.1	
Cravello et al. (2009)	Italy	human psychopharmacology	VEN (25) vs FLU (25)	VEN 17±4.5	VEN 56%	VEN 64.2±14.1	a,b
				FLU 19.2±4.4	FLU 64%	FLU 65.9±12.7	
Zhou, (2009)	China	Shandong Medical Journal	HM (45) vs FLU (45)	HM 23.15±4.11	HM 51.1%	N/A	a,b
				FLU 24.02±4.38	FLU 46.6%		
Wu, (2009)	China	Chinese Journal of Practical Nervous Disease	SER (42) vs.AMI (42)	SER 25.4±3.1	40.4%	63.2±6.5	a,c
				AMI 24.8±4.6			
Chen, (2009)	China	Guiding Journal of Traditional Chinese Medicine and Pharmacy	HM (40) vs FLU (40)	HM 24.24±6.27	HM 40%	HM 63.4±10.6	a,c
				FLU 27.35±5.68	FLU 45%	FLU 72.0±6.8	
Wang, (2009)	China	Chinese Community Doctors	PAR (55) vs.PLA (55)	PAR 23.3±6.7	31.8%	56±15	a,b,c
				PLA 22.8±7.8			
Wu, (2010)	China	Acupuncture Research	ACU (150) vs.FLU (150)	ACU 29.4±5.6	ACU 52.6%	ACU 56.2±9.2	b,c
				FLU 28.7±6	FLU 54%	FLU 55.7±9.4	
Nie et al. (2011)	China	Chinese Acupuncture	ACU (33) vs.FLU (30)	ACU 28.37±4.34	ACU 48.57%	ACU 64.2±9.85	a,c
				FLU 27.48±4.67	FLU 42.85%	FLU 63.1±9.55	
Huang et al. (2011)	China	Health industry in China	HM (30) vs FLU (30)	HM 19.6±4.2	HM 63.3%	N/A	a,c
				FLU 19.3±4.4	FLU 66.6%		
Li et al. (2011)	China	Chinese Journal of Practical Nervous Disease	DUL (45) vs.AMI (45)	DUL 29.55±2.78	DUL 33.3%	DUL 59.5±3	a,b,c
				AMI 29.8±3.26	AMI 35.5%	AMI 59.6±4.8	
Wang et al. (2011)	China	Chinese Journal of Medicinal Guide	MIR (42) vs.AMI (42)	MIR 25.4±3.1	40.4%	63.2±6.5	a,c
				AMI 24.8±4.6			
Li and Chi, (2011)	China	Chinese Acupuncture	ACU (23) vs FLU (20)	ACU 24.8±5	ACU 47.8%	ACU 56.7±14.4	a
				FLU 26.7±4.6	FLU 50%	FLU 59.4±14.1	
Shao and Qing, (2012)	China	Chinese Journal of Hospital Pharmacy	AMI (66) vs.ESC (66)	AMI 22.0±4.7	N/A	N/A	a
				ESC 22.5±4.9			
Chen et al. (2012)	China	Zhejiang Praetical MMicine	DUL (48) vs.SER (48)	DUL 23.21±5.15	DUL37.5%	DUL 63±5.56	a,b,c
				SER 23.56±4.37	SER 41.6%	SER 65±4.78	
Li and Chen, (2012)	China	Guide of China Medicine	CIT (53) vs.SER (53)	CIT 29.59±1.76	N/A	N/A	a,b
				SER 29.76±1.65			
Liu and Yan, (2012)	China	China Modern Doctor	CIT (40) vs.VEN (40)	CIT 33.6±4.2	CIT 47.5%	CIT 62.7±8.7	a,c
				VEN 32.3±4.6	VEN 42.5%	VEN 61.3±7.7	
Wu et al. (2013)	China	Hei long jiang medical journal	ESC (40) vs.PAR (40)	ESC 25.48±2.7	ESC 40.0%	ESC 67.5±9.3	a,b,c
				PAR 25.28±2.75	PAR 47.5%	PAR 68.2±11.2	
Nie and Huang, (2013)	China	Chinese Acupuncture	ACU (42) vs PAR (40)	ACU 30.37±4.24	ACU 54.7%	ACU 64±10	a,c
			vs ACU + PAR (41)	PAR 31.46±4.17	PAR 55%	PAR 64±10	
				ACU + PAR 31.35±4.07	ACU + PAR 58.5%	ACU + PAR 63±10	
Huang et al. (2014)	China	Journal of Practical Cardio-cerebral pulmonary vascular diseases	VEN (100) vs.ESC (100)	VEN 30.76±4.19	VEN 42%	VEN 69.2±2.5	a,b
				ESC 29.61±4.25	ESC 40%	ESC 69.2±2.1	
Li, (2014a)	China	Chinese Journal of Contemporary Medicine	VEN (45) vs AMI (46)	VEN 28.8±6.2	N/A	N/A	a,b,c
				AMI 29.1±6.3			
Li, (2014b)	China	Chin J Mod Drug Appl	PAR (40) vs.ESC (40)	PAR 27.06±4.09	PAR 57.5%	PAR 38.64±5.93	a,b,c
				ESC 27.22±3.99	ESC 62.5%	ESC 38.91±5.73	
Wei et al. (2014)	China	Chin J of Clinical Rational Drug Use	CIT (40) vs.AMI (40)	CIT 22.7±2.6	CIT 32.5	CIT 70.1±6.9	a
				AMI 22.3±2.8	AMI 37.5%	AMI 69.2±7.3	
Li and Yuan, (2015)	China	Frontier Journal of Medicine	MIR (42) vs.AMI (42)	MIR 30.11±3.92	MIR 42%	MIR 58±2.6	a,b
				AMI 41.41±5.22	AMI 42%	AMI 56±3.8	
Yan et al. (2015)	China	J Phys Ther Sci	HBO (30) vs FLU (30)	HBO 20.1±5.7	HBO 46.6%	HBO 63±8.1	a,c
			vs HBO + FLU (30)	FLU 19.8±4.5	FLU 43.3%	FLU 65±7.9	
				HBO + FLU 22.8±3.3	HBO + FLU 40%	HBO + FLU 66±5.9	
Wang and Qiu, (2015)	China	Anhui Medical Journal	SER (75) vs.AMI (75)	SER 31.57±3.42	SER 48%	SER 65.2±6.7	a
				AMI 32.14±4.63	AMI 45.3%	AMI 65.8±6.1	
Li, (2015)	China	Joumal of Qiqihar University of Medicine	VEN (74) vs.AMI (74)	VEN 27.6±5.4	45.9%	52.6±2.5	a,b,c
				AMI 26.3±5.7			
Chen et al. (2015)	China	Journal of International Psychiatry	CIT (48) vs PLA (48)	CIT 26.6±6.7	CIT 47.9%	CIT 60.8±9.6	b
				PLA 27.4±7	PLA 45.8%	PLA 61.0±9.5	
Feng, (2015)	China	J Clin Psychosom Dis	ESC (40) vs.SER (40)	ESC 25.47±2.71	ESC 42.45%	ESC 65±4.5	a,b,c
				SER 25.29±2.76	SER 47.5%	SER 65.3±5.1	
Sun et al. (2015a)	China	Chinese Acupuncture	ACU (31) vs FLU (31)	ACU 23.78±5.46	ACU 51.6%	ACU 67±4	a,c
			vs ACU + FLU (31)	FLU 24.23±6.16	FLU 51.6%	FLU 69±5	
				ACU + FLU 23.62±6.23	ACU + FLU 54.8%	ACU + FLU 68±5	
Sun et al. (2015b)	China	Chinese Acupuncture	PAR (30) vs ACU + PAR (33)	PAR 29.34±10.22	PAR 50%	PAR 58±8	a,c
				ACU + PAR 29.25±9.73	ACU + PAR 36.4%	ACU + PAR 59±7	
Song et al. (2016)	China	Chinese Journal of Trauma and Disability Medicine	MIR (46) vs.CIT (45)	MIR 23.73±2.07	MIR 45.6%	MIR 63.2±5.1	a,c
				CIT 22.9400B1ye1.98	CIT 48.8%	CIT 62.9±5.7	
Li, (2016)	China	China Journal of Pharmaceutical Economics	PAR (103) vs.AMI (103)	PAR 28±5	PAR 40.7%	PAR 68.5±2.1	a,b,c
				AMI 27±4	AMI 36.8%	AMI 66.54±1.7	
Hu et al. (2016)	China	Chinese Journal of Integrative Medicine on Cardio	MIR (40) vs.AMI (40)	MIR 20.96±4.52	MIR 40%	MIR 61.1±5.6	a
				AMI 20.47±3.65	AMI 37.5%	AMI 60.7±6.1	
Wu et al. (2016)	China	Chinese Journal of Practical Nervous Disease	VEN (44) vs.ESC (44)	VEN 29.8±3.6	VEN 45.4%	VEN 72.8±10.1	a,b,c
				ESC 30.6±4.4	ESC 47.5%	ESC 74.6±10.4	
Feng, (2016)	China	Chinese Journal of Practical Nervous Disease	PAR (57) vs SER (58)	PAR 23.6±2.5	PAR 45.6%	PAR 60.7xyd±6.3	b,c
				SER 23.5±2.5	SER 44.8%	SER 60.6±6.6	
Li et al. (2017c)	China	China Pharmacy	SER (42) vs PAR (49)	SER 24.75±6.03	SER 42.8%	SER 68.4±9.1	b,c
				PAR 24.46±5.89	PAR 28.5%	PAR 66.7±8.5	
Hu et al. (2017)	China	Capital Journal of Food and Medicine	ESC (50) vs MIR (50)	ESC 30.17±6.06	ESC 40%	ESC 56.42±5.18	b
				MIR 31.36±5.27	MIR 44%	MIR 54.21±6.32	
Li et al. (2017a)	China	Journal of Traditional Chinese Medicine	ACU (29) vs FLU (29)	ACU 19±7	ACU 44.8%	ACU69±7	a,b
				FLU 20±8	FLU 41.3%	FLU 68±7	
Li et al. (2017b)	China	Journal of Hebei Medical University	ESC (64) vs.PLA (62)	ESC 21.83±1.46	ESC 34.3%	ESC 62.02±9.8	a
				PLA 21.62±1.41	PLAV35.4%	PLA 62.35±9.31	
Wang et al. (2017)	China	Practical geriatrics	ESC (40) vs.PAR (40)	ESC 29.55±4.21	ESC 42.5%	ESC 67.58±6.08	a,b,c
				PAR 29.82±4.53	PAR 45%	PAR 67.15±5.95	
Lin et al. (2017)	China	Chinese Acupuncture and Moxibustion	ACU (30) vs.PAR (30)	ACU 26.72±7.63	ACU 56.6%	ACU 59±9	b,c
				PAR 25.97±8.16	PAR 53.3%	PAR 58±8	
Ye and Ye, (2018)	China	China Modern Doctor	SER (46) vs AMI (46)	SER 27.98±3.31	SER 47.8%	SER 70.08±6.41	b
				AMI 28.18±4.08	AMI 43.4%	AMI 69.81±6.45	
Chen et al. (2018)	China	Chinese Acupuncture	PLA (30) vs ACU (30)	PLA 36.6±9.5	PLA 56%	PLA 58±11	a
				BEL 35.7±10	ACU 53.3%	ACU 57±11	
Bai et al. (2018)	China	Guizhou Medical Journal	PAR (75) vs.DUL (75)	PAR 23.92±1.71	PAR 45.3%	PAR 58.12±2.37	a,b,c
				DUL 23.96±1.73	DUL 48%	DUL 59.13±2.35	
Chen and Li, (2018)	China	Medicine	PAR (35) vs.PLA (35)	PAR 27.9±6.8	PAR 34.3%	PAR 62.5±11.4	b
				PLA 26.0±5.9	PLA 42.9%	PLA 64.1±12.3	
Yang et al. (2019)	China	Yunnan Medical Journal	rTMS + PAR (49)	rTMS + PAR 21.47±1.56	rTMS + PAR 42.8%	rTMS + PAR 58.42±7.16	b,c
			vs PAR (49)	PAR 22.25±1.49	PAR 40.8%	PAR 58.72±6.94	
Cao et al. (2020)	China	Restorative Neurology and Neuroscience	CIT (52) vs PLA (47)	CIT 12.23±3.12	N/A	N/A	a,b
				PLA 11.98±3.41			
Liu, (2020)	China	Journal of reflexology and Rehabilitation Medicine	rTMS + PAR (34)	rTMS + PAR 25.22±1.25	rTMS + PAR 44.12%	rTMS + PAR 54.92±11.8	b,c
			vs PAR (34)	PAR 26.09±0.29	PAR 35.29%	PAR 55.2±12.6	
Hu et al. (2020)	China	Journal of Chinese Contemporary Medicine	rTMS + PAR (40)	rTMS + PAR 28.5±5.0031	rTMS + PAR 37.5%	rTMS + PAR 48.7±5.4	b
			vs PAR (40)	PAR 28.1±5.3	PAR 42.5%	PAR 49.3±5.8	
Wang et al. (2020)	China	Neural Injury And Functional Reconstruction	rTMS + PAR (76)	rTMS + PAR 28.34±5.13	rTMS + PAR 59.2%	rTMS + PAR 68.52±5.71	b,c
			vs PAR (76)	PAR 27.25±5.01	PAR 56.5%	PAR 68.39±5.02	
You et al. (2020)	China	Journal of Traditional Chinese Medicine	ACU + FLU (35) vs FLU (35)	N/A	38%	N/A	a
Huang et al. (2021)	China	World Journal of Integrated Traditional and Western Medicine	ACU (30) vs FLU (30)	ACU 11.89 ± 2.73	ACU 50%	ACU 63.51±6.09	a,c
				FLU 12.07±3.08	FLU 43.3%	FLU 64.14±5.47	

PLA: Placebo; ACU: Acupuncture; FLU: Fluoxetine; PAR: Paroxetine; CIT: Citalopram; rTMS: repetitive transcranial magnetic stimulation; HBO: Hyperbaric oxygen; VEN: Venlafaxine; HM: Herb medicine; AMI: amitriptyline; ESC: Escitalopram; SER: Sertraline; DUL: Duloxetine; MIR: Mirtazapine; N/A: not application.

**FIGURE 2:**
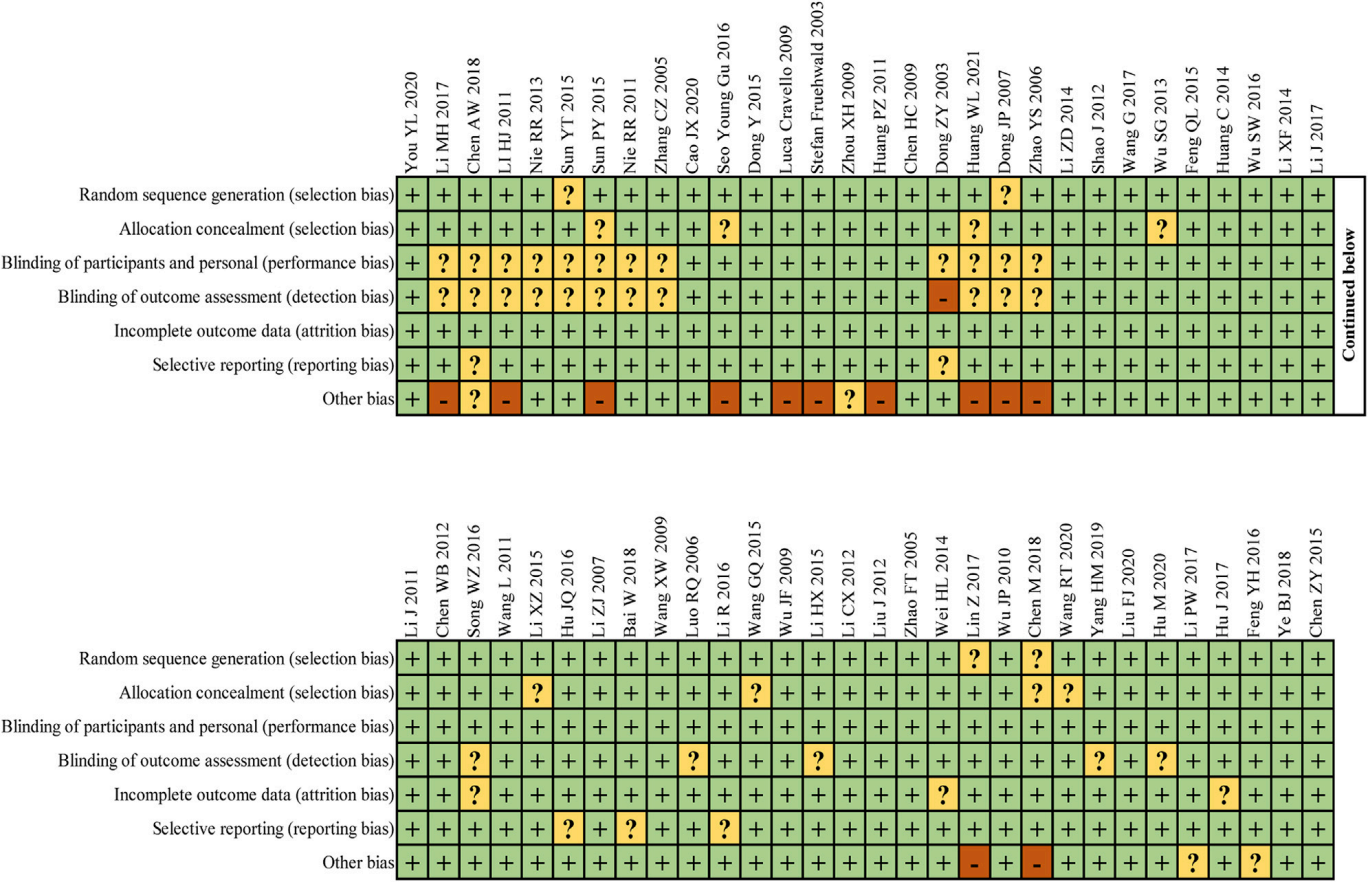
I Risk of bias summary: review authors' judgements about each risk of bias item for each included study.

In addition, we conducted a detailed bias analysis of these 60 articles. The main bias focused on the possible blinding bias associated with acupuncture and its combination therapy, which could not be avoided during their implementation. The remaining factors that may have influenced the bias focused on the number of included populations, marked in the “other bias” item. All articles marked in yellow or red, present too small included populations, with less than 25 people included in each group in Li HJ’s literature, which may have had some impact on the results (Figure 2).

### Heterogeneity examination

Before constructing the final network model, we verified the heterogeneity of the collected data, and we used a random-effects model for the two indicators of HAMD change at the end of week 4 and 8 with an overall *I* ^*2*^ > 50%. Correspondingly, the proportion of patients with 50% improvement in HAMD, an indicator for which we used a fixed-effects model to construct the network because its overall *I* ^*2*^ ≤ 50%. More detailed inter-article comparisons are shown in the supplementary materials (Supplementary Figure S1).

### Network meta-analysis

We analyzed the efficacy of different treatments for PSD, including medications, traditional herbal treatments, and medications combined with acupuncture, HBO and rTMS, by means of a network meta-analysis. We selected two indicators, including the amount of HAMD changes (including results at the end of week 4 and 8 of treatment) and the percentage of patients with 50% improved HAMD.

**FIGURE 3:**
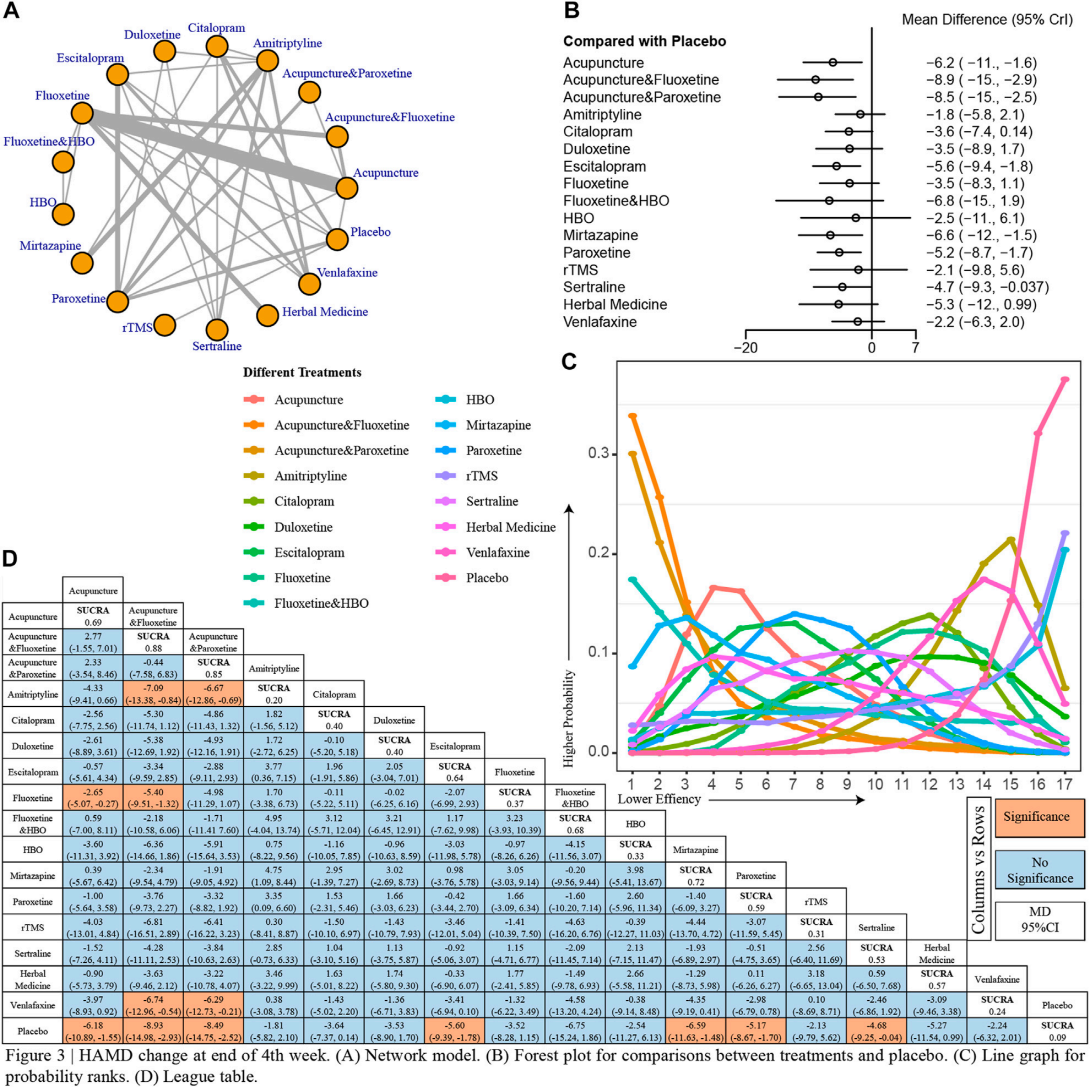
I HAMD change at end of 4th week. **(A)** Network model. **(B)** Forest plot for comparisons between treatments and placebo. **(C)** Line graph for probability ranks. **(D)** League table.

Totally 17 treatments were enrolled in the analysis of the changes in HAMD score at the end of week 4 (Figure 3A). At the end of the 4th week of treatment, acupuncture (MD, −6.2; 95% CI, [−11, −1.6]), acupuncture combined with fluxetine (MD, −8.9; 95% CI, [−15, −2.9]), acupuncture combined with paroxetine (MD,—8.5; 95% CI, [−15, −2.5]), escitalopram (MD, −5.6; 95% CI, [−9.4, −1.8]), mirtazapine (MD, −6.6; 95% CI, [−12, −1.5]), paroxetine (MD, −5.2; 95% CI, [−8.7, −1.7]), and sertraline (MD, −4.7; 95% CI, [−9.3, −0.037]) were significantly superior compared to placebo (Figure 3B). Based on the evidence from the network, we drew line graphs showing the rank of probability for each treatment (Figure 3C). The results of the line graph suggest that acupuncture combined with fluoxetine was the most effective treatment at the end of the 4th week while the placebo was clearly the worst. More specific inter-treatment comparisons we show in the league table (Figure 3D). It can be found that acupuncture combined with fluoxetine is more effective to amitriptyline, venlafaxine, and placebo for PSD. Of interest is that the efficacy of fluoxetine after combined acupuncture is better than that of fluoxetine alone (MD, −5.4; 95% CI, [−9.51, −1.32]), which is statistically significant.

**FIGURE 4:**
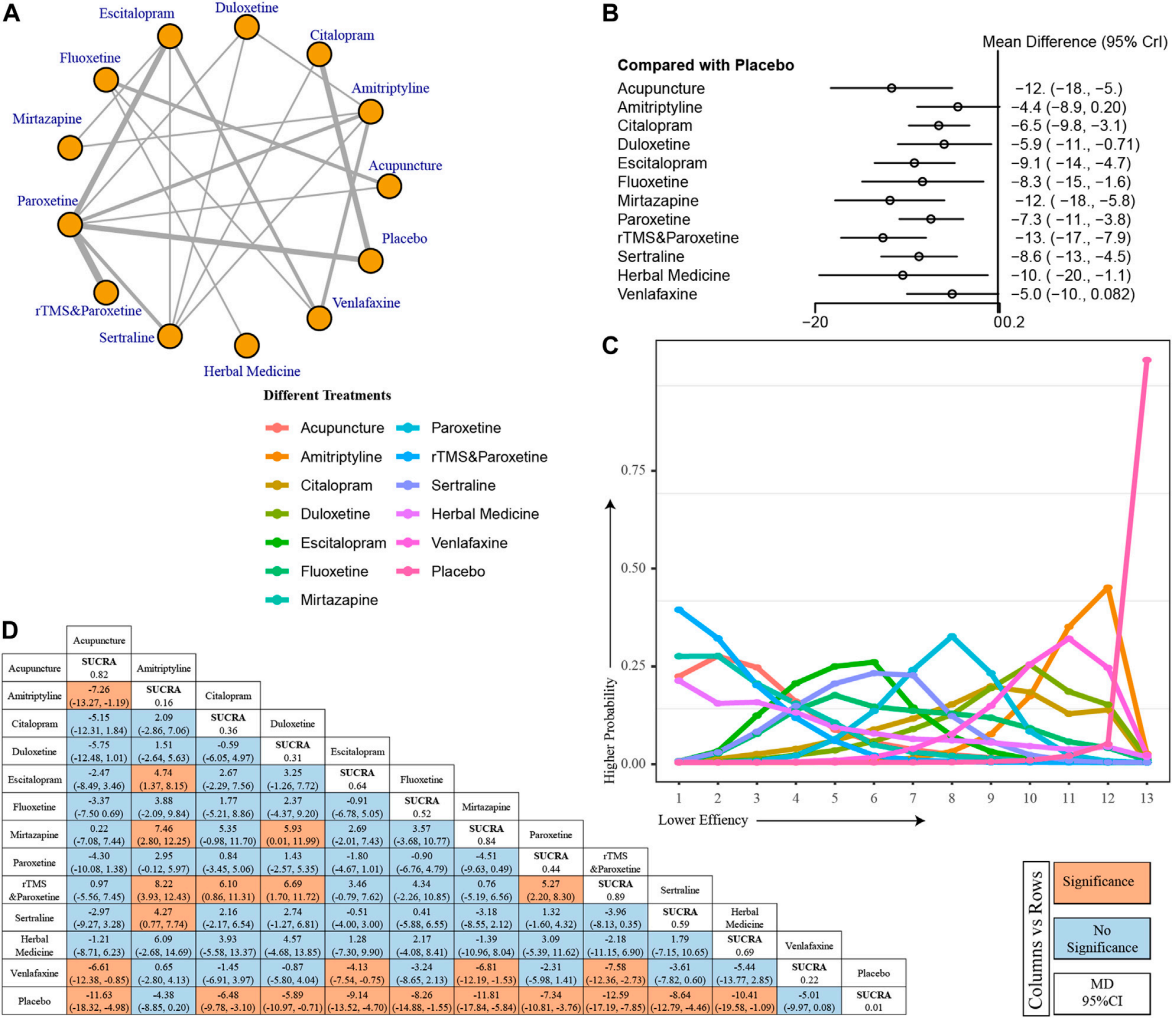
I HAMD change at end of 8th week. **(A)** Network model. **(B)** Forest plot for comparisons between treatments and placebo. **(C)** Line graph for probability ranks. **(D)** League table.

When the course of treatment was extended to 8 months, 13 modalities were included in our analysis (Figure 4A). All treatments showed better improvement compared to placebo, except amitriptyline (MD, −4.4; 95% CI, [−8.9, −0.2]), venlafaxine (MD, −5; 95% CI, [−10, −0.082]) (Figure 4B). Also, by inspection of the line graph of probability rank, we found that paroxetine treatment in combination with rTMS was the most effective in improving PSD at the end of the 8th week (Figure 4C). There is no doubt that the placebo group had the worst effect. Using the detailed data from the league table, we found that acupuncture is still an effective treatment modality, compared to placebo. Similarly, we found that paroxetine treatment in combination with rTMS was superior to paroxetine (rTMs and paroxetine vs paroxetine MD, −5.27; 95% CI, [−2.2, −8.3]) treatment alone (Figure 4D).

**FIGURE 5:**
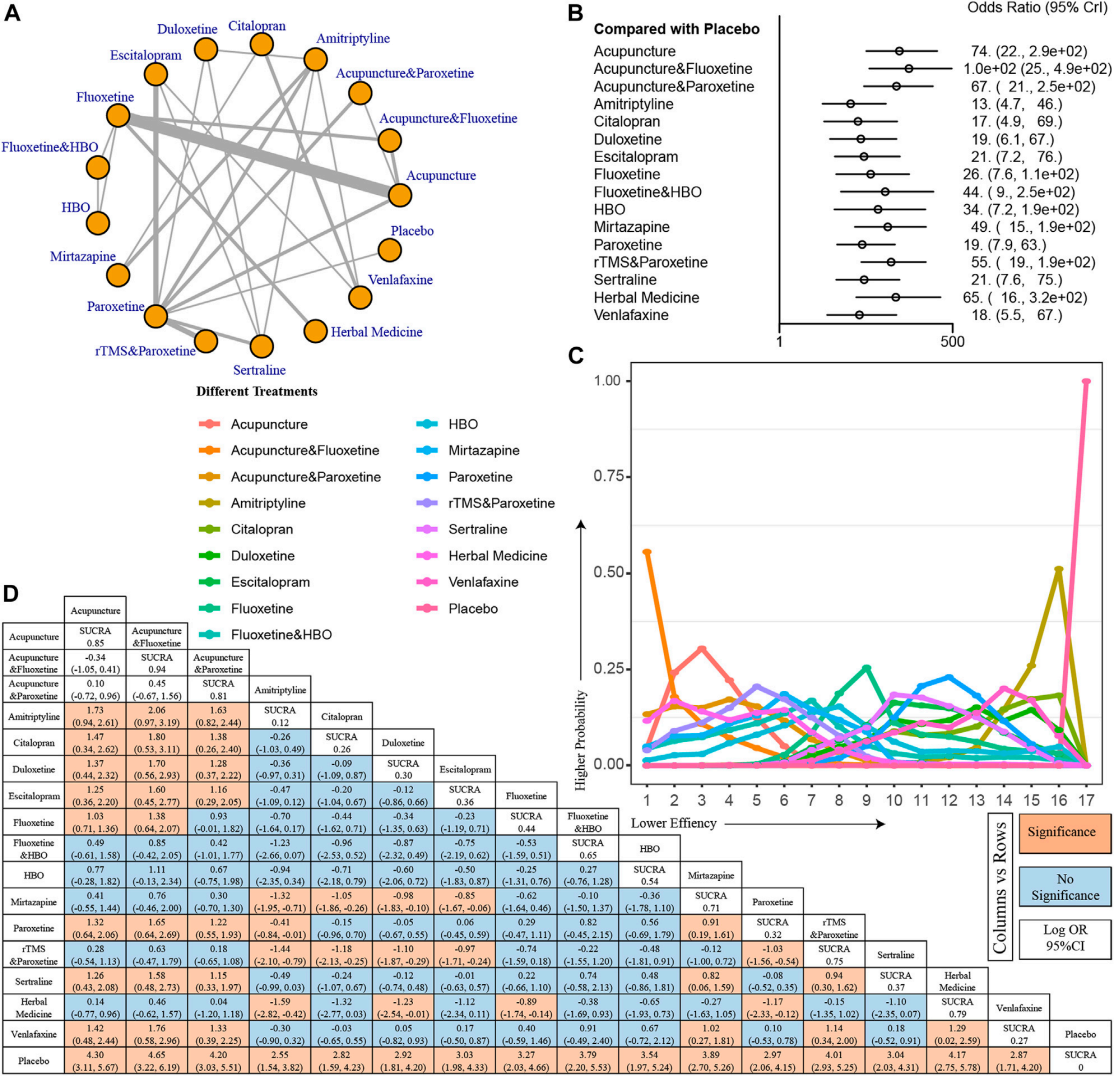
I Percentage of patients with 50% improvement in HAMD. **(A)** Network model. **(B)** Forest plot for comparisons between treatments and placebo. **(C)** Line graph for probability ranks. **(D)** League table.

Seventeen treatments were included for comparison to assess the impact of different treatments on the percentage of patients with 50% improvement in HAMD (Figure 5A). All included treatment modalities demonstrated a better 50% improvement rate than placebo. Several combination treatment modalities, acupuncture combined with fluoxetine (OR, 1.0e+02; 95% CI, [25, 4.9e+02]), acupuncture combined with paroxetine (OR, 67; 95% CI, [21, 2.5e+02]), HBO combined with fluoxetine (OR, 44; 95% CI, [9, 2.5e+02]), and rTMS combined with paroxetine (OR, 55; 95% CI, [19, 1.9e+02]) showed good efficacy compared to placebo (Figure 5B). The efficacy of acupuncture combined with fluoxetine was the best in the line chart, and the efficacy of acupuncture combined with paroxetine and rTMS combined with paroxetine also ranked high (Figure 5C). Data from the league table also suggest that acupuncture combined with fluoxetine and rTMS combined with paroxetine is more effective than traditional antidepressants such as citalopram, amitriptyline, and duloxetine alone (Figure 5D).

### Consistency and similarity examinations

Direct, indirect, and pooling evidence are available in the network for all indicators. Therefore, for each indicator, we used the node-splitting method to perform consistency tests. All 3 indicators were not tested for inconsistency between indirect and direct evidence, with all *p*-values >0.05, suggesting that our model was constructed reasonably correctly (Supplementary Figure S2). Similarity tests were performed by the bias analysis and quality control of the included articles. There was no significant bias in similarity between data, as previously described.

## Discussion

PSD is the most common mental health problem, afflicting approximately 33% of stroke survivors, and PSD affects the recovery and quality of life of post-stroke patients (Robinson and Jorge, 2016). A growing amount of research suggests a strong association between PSD and stroke, and that both physiology and psychology play an important role in PSD (Zhou et al., 2022). Despite extensive efforts to study PSD, its pathogenesis is still unclear. Currently, selective 5-hydroxytryptamine reuptake inhibitors and tricyclic antidepressants are the main treatments in clinical practice (Paolucci, 2017). In recent years, there are more and more new treatment strategy for PSD, such as acupuncture, herbal medicine, HBO and rTMS (Hadidi et al., 2017). However, it is not clear which of the different treatments is more effective. In our study, we performed a Bayesian network meta-analysis of the 60 included articles to compare the effectiveness of different treatment methods. Our study found that the combination of antidepressants with adjuvant therapy enhanced the efficacy of antidepressants and was superior to antidepressant monotherapy. This provides some theoretical basis for future clinical work.

In this paper, we constructed an appropriate network meta-analysis model by collecting data from more than a dozen treatment modalities from previous works to analyze 3 indicators of effectiveness of PSD. The treatments we enrolled included commonly used antidepressant medications, herbal medicine, HBO, acupuncture, and medications combined with adjuvant therapy, covering almost all current clinical applications for the treatment of PSD. All 3 analyzed indicators were related to the HAMD score, the most widely used scale to evaluate the degree of depression in patients. The HAMD is a 17-item clinical assessment instrument used to quantify the severity of depression in subjects who have been diagnosed with depression (Hamilton, 1967). It became one of the most widely used prognostic indicators in depression and was used in many trials of new antidepressant drugs submitted to the Food and Drug Administration (FDA) (Leucht et al., 2013). We studied here only the amount of HAMD change at the 4th and 8th week end, and the percentage of patients with 50% improvement in HAMD, with the intention of analyzing which treatment modality is the most efficacious now.

Our analysis pointed out that at the end of the 4th week, not all classical antidepressants produced an effect, with only escitalopram, paroxetine, mirtazapine, and sertraline having significant efficacy. Antidepressants, including selective 5-hydroxytryptamine reuptake inhibitors and tricyclic antidepressants, have been shown to be effective in PSD. Although there are no studies showing that selective 5-hydroxytryptamine reuptake inhibitors are more effective than tricyclic antidepressants, selective 5-hydroxytryptamine reuptake inhibitors are currently used as the first line of treatment. This may be due to the fact that selective 5-hydroxytryptamine reuptake inhibitors is more effective in treating depression and generalized anxiety disorder and has fewer long-term side effects (Qin et al., 2014; Villa et al., 2018). In previous studies, tricyclic antidepressants, such as amitriptyline, were found to take 2–3 weeks to exert their effects and selective 5-hydroxytryptamine reuptake inhibitors took 3–4 weeks to work (Gillman, 2007). Therefore, the amount of change in HAMD scores at the fourth week after treatment may not be significant. And the treatments combined with acupuncture greatly enhanced the therapeutic effect of antidepressants. Among them, fluoxetine combined with acupuncture treatment has the greatest possibility to produce the best efficacy, according to our calculations. It was significantly superior compared to fluoxetine alone, initially demonstrating the effect of the combination treatment. The possible mechanism of acupuncture for the treatment of PSD is achieved by stimulating specific acupoints to induce the restoration of brain nerve cells, including noradrenergic, dopamine, 5-hydroxytryptamine neurons and their axons, while accelerating the repair of damaged brain tissue and promoting the release of 5-hydroxytryptamine in the brain and noradrenaline in the spinal cord (Zhang et al., 2010; Li et al., 2020) The mechanism by which herbs and rTMS exert their antidepressant effects is not fully understood. However, studies have shown that they can increase blood flow to local lesions, improve cortical metabolism, promote neuronal protein expression, and thus improve depressive symptoms (Liu et al., 2012; Liu et al., 2015). Therefore, drug treatment combined with acupuncture, rTMS and herbal medicine can have better therapeutic effect to a certain extent.

When the time window of treatment was stretched to 8 weeks, all treatment modalities except amitriptyline and griseofulvin achieved good efficacy. Notably, rTMS combined with paroxetine was most likely to achieve the most excellent treatment results at this time. Once again, the efficacy of the combination therapy exceeded that of paroxetine monotherapy. The better treatment outcome may be due to the longer treatment period compared to 4 weeks of treatment.

For overall effectiveness, the metric we chose was the percentage of patients with 50% improvement in HAMD. Our results continue to point to a preference for the use of combination therapy that can improve the depressive status of patients. Both acupuncture combined with fluoxetine and acupuncture combined with paroxetine, which performed well at the end of 4 weekends, and rTMS combined with paroxetine, which performed well at the end of 8 weekends, were in the top 5 most effective. This further confirms the good effect of the combined treatment. In contrast, we noted that while amitriptyline and venlafaxine had a higher percentage of patients with HAMD improvements of more than 50% than placebo. However, neither antidepressant showed better improvement than the placebo group at the end of 4 and 8 weeks. This may have a negative impact on their continued use as PSD medication in the future. Previous studies have shown that amitriptyline and venlafaxine have better treatment effects than placebo when used to treat depression. This result is inconsistent with the results of our study (Rampello et al., 1995; Furukawa et al., 2019). This may be since PSD has a different pathogenesis than common depression. For example, the PSD-related potentials are different from common depression-related potentials (Hajcak et al., 2010). This differences leads to the poor therapeutic effect of amitriptyline and venlafaxine in the treatment of PSD. The differences in their clinical effects need to be further investigated.

Although we concluded the above results, there are some limitations in the paper that may have an impact on our results. First, we have only conducted a limited analysis of the effectiveness of these treatments and lack relevant comparisons in terms of safety and other aspects. Although many treatments showed significant improvements in PSD, their side effects may cause adverse events in patients beyond depression. Second, the article included a limited population and a large sample of Chinese patients was included in the article, which may have introduced bias to the included population. The third point is that the included articles do not strictly distinguish between hemorrhagic or ischemic stroke. Both may have an impact on the way and severity of PSD. In addition, inconsistencies in the treatment modalities included at the end of week 4 and 8 are due to problems inherent in the data sources. Some treatment modalities were missing, making it impossible to compare the efficacy of the same treatment modality at different times. The fifth point is that only the HAMD score was used as the primary outcome indicator in this study, which would have missed some of the articles using other outcome indicators, such as geriatric depression scale and hospital anxiety and depression scale. Finally, there are many traditional Chinese medicines for the treatment of post-stroke depression, such as xiaoyaosan, shuganjieyutang and sinisan. However, in this study, only shuganjieyutang was included for meta-analysis because other medication outcome indicators were inconsistent or did not meet the inclusion criteria of this study.

## Conclusion

In conclusion, for HAMD change effects at the end of the 4th and 8th week of PSD treatment, the combination of antidepressants with adjuvant therapy may enhance the efficacy of antidepressants and achieve better results than antidepressant monotherapy. According to our findings, acupuncture combined with fluoxetine treatment was more effective in the treatment of post-stroke depression at week 4 and had higher 50% HAMD improvement rate, while rTMS combined with paroxetine was more effective at week 8. Further research is needed to determine whether acupuncture combined with fluoxetine is better than rTMS combined with paroxetine for post-stroke depression at week 8. All conclusions still need to be justified by further clinical data.

## Data Availability

The original contributions presented in the study are included in the article/Supplementary Material, further inquiries can be directed to the corresponding authors.

## Abbreviations

RCT, randomized controlled trial; OR, odds ratio; MD, mean difference; SUCRA, surface under the cumulative ranking curve; CI, confidence interval.
