# Supplementary material for: Treatments to post-stroke depression, which is more effective to HAMD improvement? A network meta-analysis
Source: Front Pharmacol. 2022 Dec 19;13:1035895. doi: 10.3389/fphar.2022.1035895 (PMC9806231; doi:10.3389/fphar.2022.1035895)
Supplement: Supplementary file 1 [file DataSheet1.zip › SupplementaryMaterialFigureS1.pdf]

Figure S1 | Forest plots for the heterogeneity of efficacy indicators. (A) HAMD change at end of 4th week. (B) HAMD change at end of 8th week. (C) Percentage of patients with 50% improvement in HAMD.

A

Continued from left

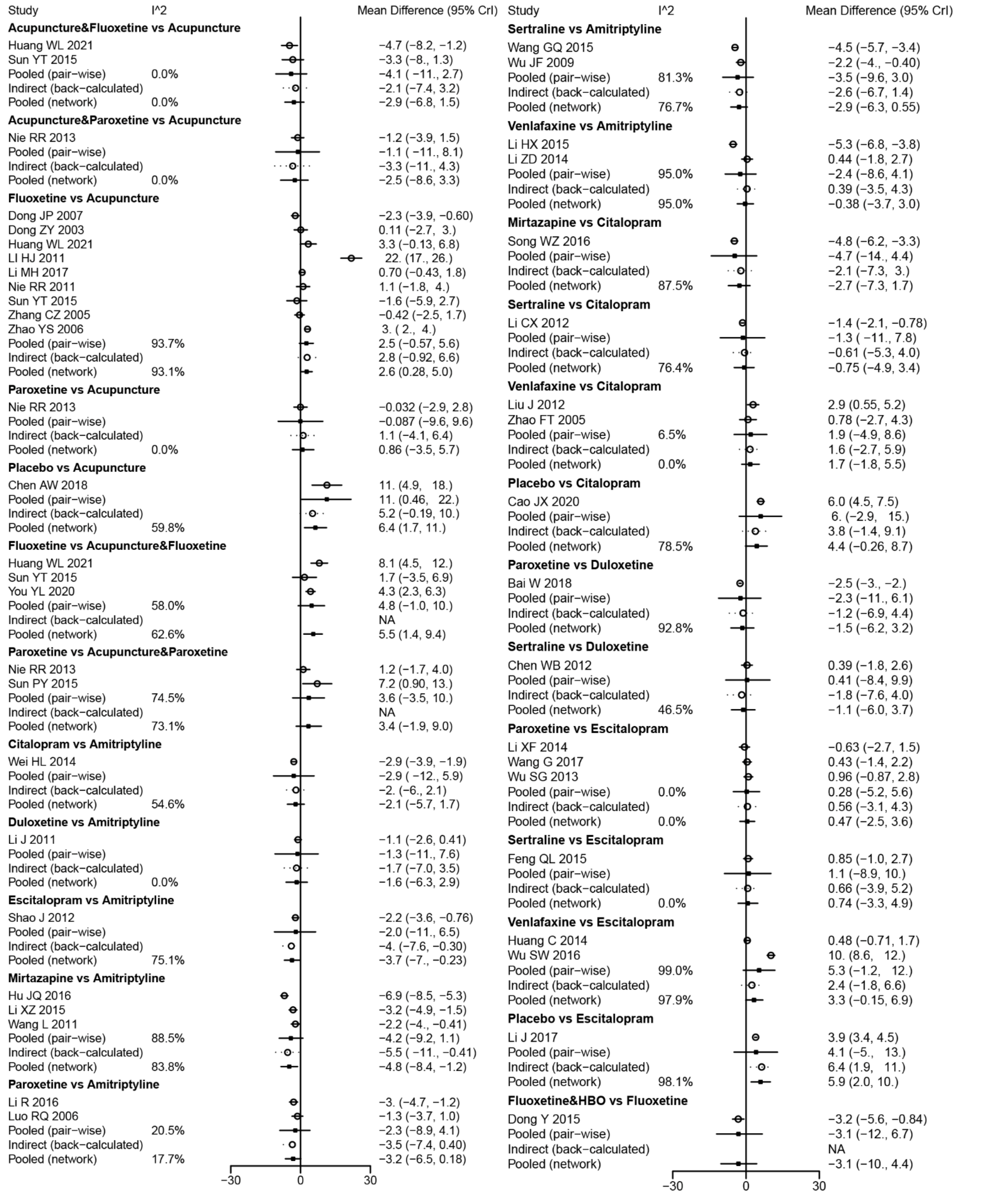

A

Continued from above

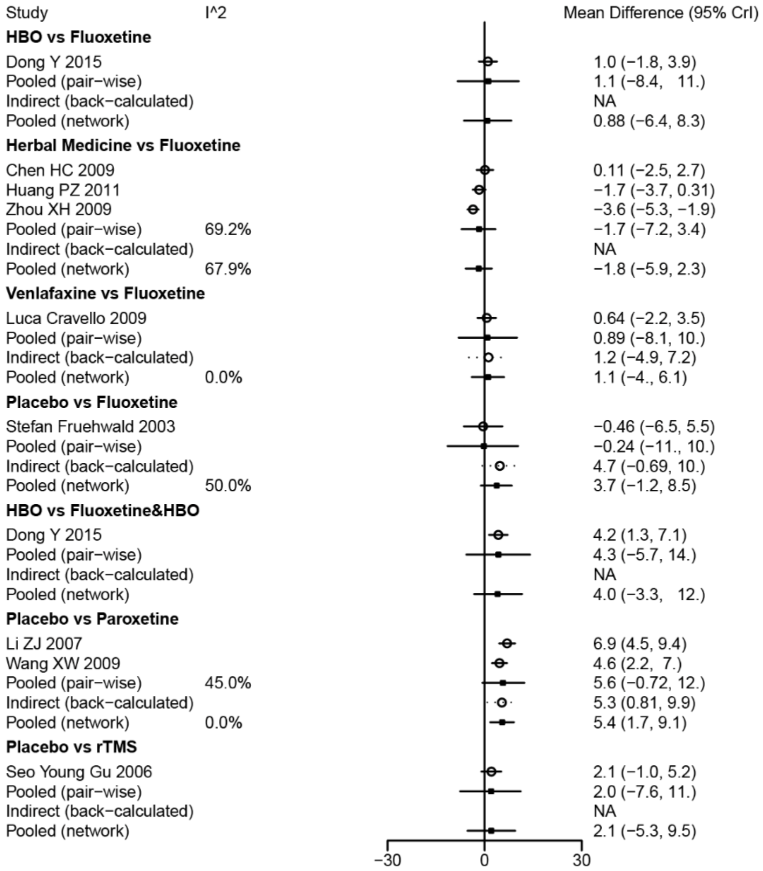

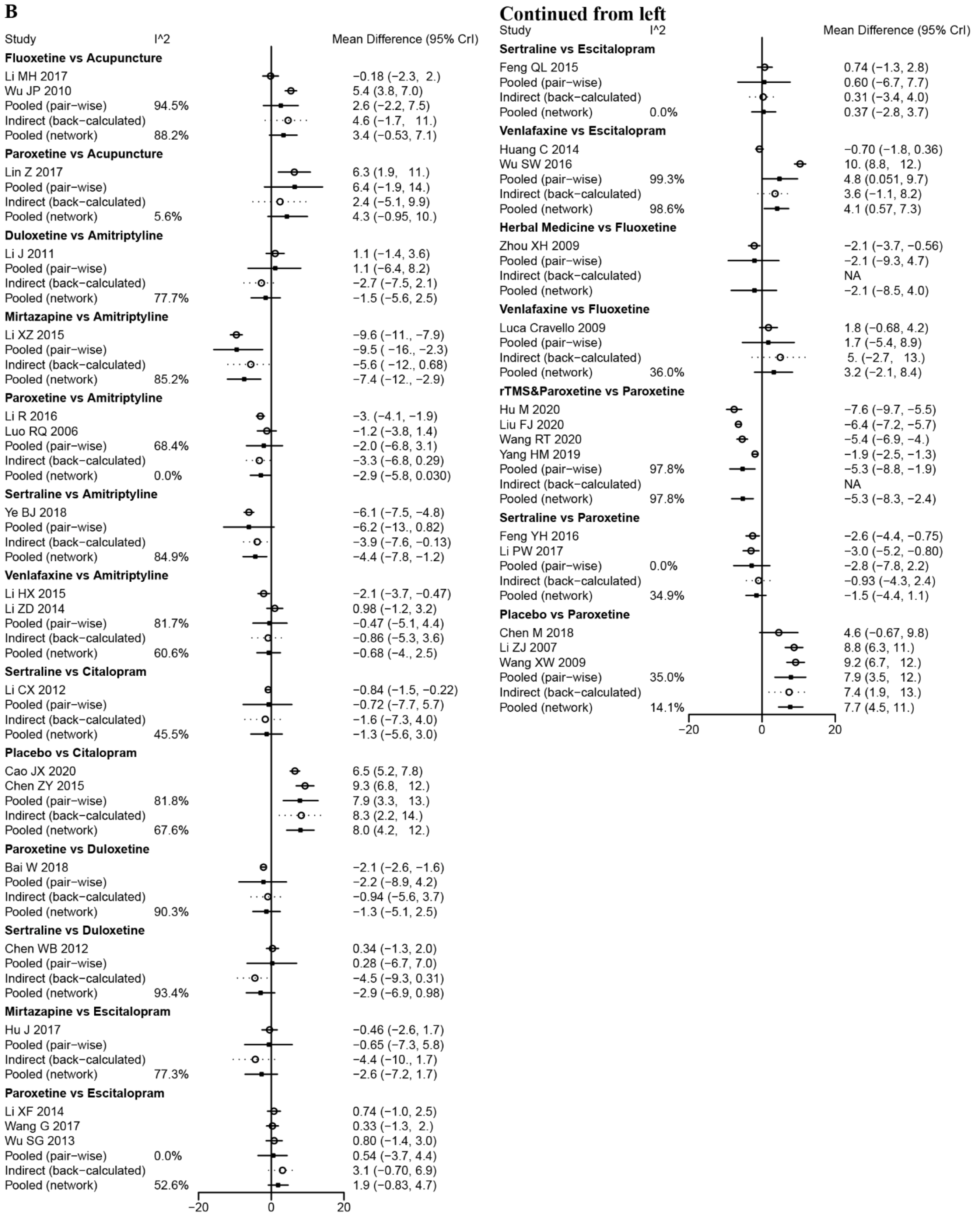

B
Study
I^2
Mean Difference (95% CrI)
Fluoxetine vs Acupuncture
Li MH 2017 −0.18 (−2.3, 2.)
Wu JP 2010 5.4 (3.8, 7.0)
Pooled (pair−wise) 94.5% 2.6 (−2.2, 7.5)
Indirect (back−calculated) 4.6 (−1.7, 11.)
Pooled (network) 88.2% 3.4 (−0.53, 7.1)
Paroxetine vs Acupuncture
Lin Z 2017 6.3 (1.9, 11.)
Pooled (pair−wise) 6.4 (−1.9, 14.)
Indirect (back−calculated) 2.4 (−5.1, 9.9)
Pooled (network) 5.6% 4.3 (−0.95, 10.)
Duloxetine vs Amitriptyline
Li J 2011 1.1 (−1.4, 3.6)
Pooled (pair−wise) 1.1 (−6.4, 8.2)
Indirect (back−calculated) −2.7 (−7.5, 2.1)
Pooled (network) 77.7% −1.5 (−5.6, 2.5)
Mirtazapine vs Amitriptyline
Li XZ 2015 −9.6 (−11., −7.9)
Pooled (pair−wise) −9.5 (−16., −2.3)
Indirect (back−calculated) −5.6 (−12., 0.68)
Pooled (network) 85.2% −7.4 (−12., −2.9)
Paroxetine vs Amitriptyline
Li R 2016 −3. (−4.1, −1.9)
Luo RQ 2006 −1.2 (−3.8, 1.4)
Pooled (pair−wise) 68.4% −2.0 (−6.8, 3.1)
Indirect (back−calculated) −3.3 (−6.8, 0.29)
Pooled (network) 0.0% −2.9 (−5.8, 0.030)
Sertraline vs Amitriptyline
Ye BJ 2018 −6.1 (−7.5, −4.8)
Pooled (pair−wise) −6.2 (−13., 0.82)
Indirect (back−calculated) −3.9 (−7.6, −0.13)
Pooled (network) 84.9% −4.4 (−7.8, −1.2)
Venlafaxine vs Amitriptyline
Li HX 2015 −2.1 (−3.7, −0.47)
Li ZD 2014 0.98 (−1.2, 3.2)
Pooled (pair−wise) 81.7% −0.47 (−5.1, 4.4)
Indirect (back−calculated) −0.86 (−5.3, 3.6)
Pooled (network) 60.6% −0.68 (−4., 2.5)
Sertraline vs Citalopram
Li CX 2012 −0.84 (−1.5, −0.22)
Pooled (pair−wise) −0.72 (−7.7, 5.7)
Indirect (back−calculated) −1.6 (−7.3, 4.0)
Pooled (network) 45.5% −1.3 (−5.6, 3.0)
Placebo vs Citalopram
Cao JX 2020 6.5 (5.2, 7.8)
Chen ZY 2015 9.3 (6.8, 12.)
Pooled (pair−wise) 81.8% 7.9 (3.3, 13.)
Indirect (back−calculated) 8.3 (2.2, 14.)
Pooled (network) 67.6% 8.0 (4.2, 12.)
Paroxetine vs Duloxetine
Bai W 2018 −2.1 (−2.6, −1.6)
Pooled (pair−wise) −2.2 (−8.9, 4.2)
Indirect (back−calculated) −0.94 (−5.6, 3.7)
Pooled (network) 90.3% −1.3 (−5.1, 2.5)
Sertraline vs Duloxetine
Chen WB 2012 0.34 (−1.3, 2.0)
Pooled (pair−wise) 0.28 (−6.7, 7.0)
Indirect (back−calculated) −4.5 (−9.3, 0.31)
Pooled (network) 93.4% −2.9 (−6.9, 0.98)
Mirtazapine vs Escitalopram
Hu J 2017 −0.46 (−2.6, 1.7)
Pooled (pair−wise) −0.65 (−7.3, 5.8)
Indirect (back−calculated) −4.4 (−10., 1.7)
Pooled (network) 77.3% −2.6 (−7.2, 1.7)
Paroxetine vs Escitalopram
Li XF 2014 0.74 (−1.0, 2.5)
Wang G 2017 0.33 (−1.3, 2.)
Wu SG 2013 0.80 (−1.4, 3.0)
Pooled (pair−wise) 0.0% 0.54 (−3.7, 4.4)
Indirect (back−calculated) 3.1 (−0.70, 6.9)
Pooled (network) 52.6% 1.9 (−0.83, 4.7)
−20
0
20
Continued from left
Study
I^2
Mean Difference (95% CrI)
Sertraline vs Escitalopram
Feng QL 2015 0.74 (−1.3, 2.8)
Pooled (pair−wise) 0.60 (−6.7, 7.7)
Indirect (back−calculated) 0.31 (−3.4, 4.0)
Pooled (network) 0.0% 0.37 (−2.8, 3.7)
Venlafaxine vs Escitalopram
Huang C 2014 −0.70 (−1.8, 0.36)
Wu SW 2016 10. (8.8, 12.)
Pooled (pair−wise) 99.3% 4.8 (0.051, 9.7)
Indirect (back−calculated) 3.6 (−1.1, 8.2)
Pooled (network) 98.6% 4.1 (0.57, 7.3)
Herbal Medicine vs Fluoxetine
Zhou XH 2009 −2.1 (−3.7, −0.56)
Pooled (pair−wise) −2.1 (−9.3, 4.7)
Indirect (back−calculated) NA
Pooled (network) −2.1 (−8.5, 4.0)
Venlafaxine vs Fluoxetine
Luca Cravello 2009 1.8 (−0.68, 4.2)
Pooled (pair−wise) 1.7 (−5.4, 8.9)
Indirect (back−calculated) 5. (−2.7, 13.)
Pooled (network) 36.0% 3.2 (−2.1, 8.4)
rTMS&Paroxetine vs Paroxetine
Hu M 2020 −7.6 (−9.7, −5.5)
Liu FJ 2020 −6.4 (−7.2, −5.7)
Wang RT 2020 −5.4 (−6.9, −4.)
Yang HM 2019 −1.9 (−2.5, −1.3)
Pooled (pair−wise) 97.8% −5.3 (−8.8, −1.9)
Indirect (back−calculated) NA
Pooled (network) 97.8% −5.3 (−8.3, −2.4)
Sertraline vs Paroxetine
Feng YH 2016 −2.6 (−4.4, −0.75)
Li PW 2017 −3.0 (−5.2, −0.80)
Pooled (pair−wise) 0.0% −2.8 (−7.8, 2.2)
Indirect (back−calculated) −0.93 (−4.3, 2.4)
Pooled (network) 34.9% −1.5 (−4.4, 1.1)
Placebo vs Paroxetine
Chen M 2018 4.6 (−0.67, 9.8)
Li ZJ 2007 8.8 (6.3, 11.)
Wang XW 2009 9.2 (6.7, 12.)
Pooled (pair−wise) 35.0% 7.9 (3.5, 12.)
Indirect (back−calculated) 7.4 (1.9, 13.)
Pooled (network) 14.1% 7.7 (4.5, 11.)
−20
0
20

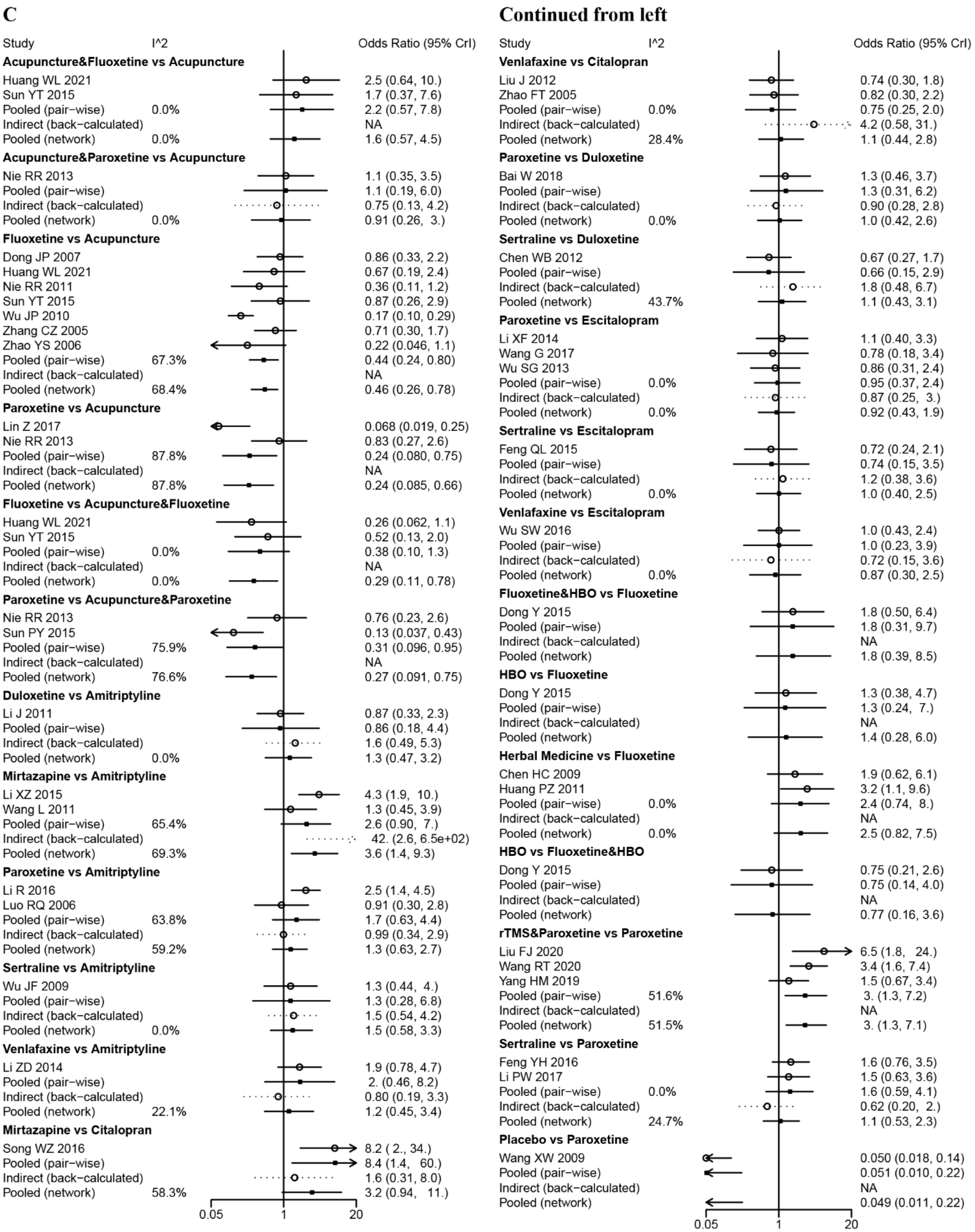
C
Study I^2 Odds Ratio (95% CrI)
Acupuncture&Fluoxetine vs Acupuncture
Huang WL 2021 2.5 (0.64, 10.)
Sun YT 2015 1.7 (0.37, 7.6)
Pooled (pair-wise) 0.0% 2.2 (0.57, 7.8)
Indirect (back-calculated) NA
Pooled (network) 0.0% 1.6 (0.57, 4.5)
Acupuncture&Paroxetine vs Acupuncture
Nie RR 2013 1.1 (0.35, 3.5)
Pooled (pair-wise) 1.1 (0.19, 6.0)
Indirect (back-calculated) 0.75 (0.13, 4.2)
Pooled (network) 0.0% 0.91 (0.26, 3.)
Fluoxetine vs Acupuncture
Dong JP 2007 0.86 (0.33, 2.2)
Huang WL 2021 0.67 (0.19, 2.4)
Nie RR 2011 0.36 (0.11, 1.2)
Sun YT 2015 0.87 (0.26, 2.9)
Wu JP 2010 0.17 (0.10, 0.29)
Zhang CZ 2005 0.71 (0.30, 1.7)
Zhao YS 2006 0.22 (0.046, 1.1)
Pooled (pair-wise) 67.3% 0.44 (0.24, 0.80)
Indirect (back-calculated) NA
Pooled (network) 68.4% 0.46 (0.26, 0.78)
Paroxetine vs Acupuncture
Lin Z 2017 0.068 (0.019, 0.25)
Nie RR 2013 0.83 (0.27, 2.6)
Pooled (pair-wise) 87.8% 0.24 (0.080, 0.75)
Indirect (back-calculated) NA
Pooled (network) 87.8% 0.24 (0.085, 0.66)
Fluoxetine vs Acupuncture&Fluoxetine
Huang WL 2021 0.26 (0.062, 1.1)
Sun YT 2015 0.52 (0.13, 2.0)
Pooled (pair-wise) 0.0% 0.38 (0.10, 1.3)
Indirect (back-calculated) NA
Pooled (network) 0.0% 0.29 (0.11, 0.78)
Paroxetine vs Acupuncture&Paroxetine
Nie RR 2013 0.76 (0.23, 2.6)
Sun PY 2015 0.13 (0.037, 0.43)
Pooled (pair-wise) 75.9% 0.31 (0.096, 0.95)
Indirect (back-calculated) NA
Pooled (network) 76.6% 0.27 (0.091, 0.75)
Duloxetine vs Amitriptyline
Li J 2011 0.87 (0.33, 2.3)
Pooled (pair-wise) 0.86 (0.18, 4.4)
Indirect (back-calculated) 1.6 (0.49, 5.3)
Pooled (network) 0.0% 1.3 (0.47, 3.2)
Mirtazapine vs Amitriptyline
Li XZ 2015 4.3 (1.9, 10.)
Wang L 2011 1.3 (0.45, 3.9)
Pooled (pair-wise) 65.4% 2.6 (0.90, 7.)
Indirect (back-calculated) 42. (2.6, 6.5e+02)
Pooled (network) 69.3% 3.6 (1.4, 9.3)
Paroxetine vs Amitriptyline
Li R 2016 2.5 (1.4, 4.5)
Luo RQ 2006 0.91 (0.30, 2.8)
Pooled (pair-wise) 63.8% 1.7 (0.63, 4.4)
Indirect (back-calculated) 0.99 (0.34, 2.9)
Pooled (network) 59.2% 1.3 (0.63, 2.7)
Sertraline vs Amitriptyline
Wu JF 2009 1.3 (0.44, 4.)
Pooled (pair-wise) 1.3 (0.28, 6.8)
Indirect (back-calculated) 1.5 (0.54, 4.2)
Pooled (network) 0.0% 1.5 (0.58, 3.3)
Venlafaxine vs Amitriptyline
Li ZD 2014 1.9 (0.78, 4.7)
Pooled (pair-wise) 2. (0.46, 8.2)
Indirect (back-calculated) 0.80 (0.19, 3.3)
Pooled (network) 22.1% 1.2 (0.45, 3.4)
Mirtazapine vs Citalopran
Song WZ 2016 8.2 (2., 34.)
Pooled (pair-wise) 8.4 (1.4, 60.)
Indirect (back-calculated) 1.6 (0.31, 8.0)
Pooled (network) 58.3% 3.2 (0.94, 11.)
0.05
1
20
Continued from left
Study I^2 Odds Ratio (95% CrI)
Venlafaxine vs Citalopran
Liu J 2012 0.74 (0.30, 1.8)
Zhao FT 2005 0.82 (0.30, 2.2)
Pooled (pair-wise) 0.0% 0.75 (0.25, 2.0)
Indirect (back-calculated) 4.2 (0.58, 31.)
Pooled (network) 28.4% 1.1 (0.44, 2.8)
Paroxetine vs Duloxetine
Bai W 2018 1.3 (0.46, 3.7)
Pooled (pair-wise) 1.3 (0.31, 6.2)
Indirect (back-calculated) 0.90 (0.28, 2.8)
Pooled (network) 0.0% 1.0 (0.42, 2.6)
Sertraline vs Duloxetine
Chen WB 2012 0.67 (0.27, 1.7)
Pooled (pair-wise) 0.66 (0.15, 2.9)
Indirect (back-calculated) 1.8 (0.48, 6.7)
Pooled (network) 43.7% 1.1 (0.43, 3.1)
Paroxetine vs Escitalopram
Li XF 2014 1.1 (0.40, 3.3)
Wang G 2017 0.78 (0.18, 3.4)
Wu SG 2013 0.86 (0.31, 2.4)
Pooled (pair-wise) 0.0% 0.95 (0.37, 2.4)
Indirect (back-calculated) 0.87 (0.25, 3.)
Pooled (network) 0.0% 0.92 (0.43, 1.9)
Sertraline vs Escitalopram
Feng QL 2015 0.72 (0.24, 2.1)
Pooled (pair-wise) 0.74 (0.15, 3.5)
Indirect (back-calculated) 1.2 (0.38, 3.6)
Pooled (network) 0.0% 1.0 (0.40, 2.5)
Venlafaxine vs Escitalopram
Wu SW 2016 1.0 (0.43, 2.4)
Pooled (pair-wise) 1.0 (0.23, 3.9)
Indirect (back-calculated) 0.72 (0.15, 3.6)
Pooled (network) 0.0% 0.87 (0.30, 2.5)
Fluoxetine&HBO vs Fluoxetine
Dong Y 2015 1.8 (0.50, 6.4)
Pooled (pair-wise) 1.8 (0.31, 9.7)
Indirect (back-calculated) NA
Pooled (network) 1.8 (0.39, 8.5)
HBO vs Fluoxetine
Dong Y 2015 1.3 (0.38, 4.7)
Pooled (pair-wise) 1.3 (0.24, 7.)
Indirect (back-calculated) NA
Pooled (network) 1.4 (0.28, 6.0)
Herbal Medicine vs Fluoxetine
Chen HC 2009 1.9 (0.62, 6.1)
Huang PZ 2011 3.2 (1.1, 9.6)
Pooled (pair-wise) 0.0% 2.4 (0.74, 8.)
Indirect (back-calculated) NA
Pooled (network) 0.0% 2.5 (0.82, 7.5)
HBO vs Fluoxetine&HBO
Dong Y 2015 0.75 (0.21, 2.6)
Pooled (pair-wise) 0.75 (0.14, 4.0)
Indirect (back-calculated) NA
Pooled (network) 0.77 (0.16, 3.6)
rTMS&Paroxetine vs Paroxetine
Liu FJ 2020 6.5 (1.8, 24.)
Wang RT 2020 3.4 (1.6, 7.4)
Yang HM 2019 1.5 (0.67, 3.4)
Pooled (pair-wise) 51.6% 3. (1.3, 7.2)
Indirect (back-calculated) NA
Pooled (network) 51.5% 3. (1.3, 7.1)
Sertraline vs Paroxetine
Feng YH 2016 1.6 (0.76, 3.5)
Li PW 2017 1.5 (0.63, 3.6)
Pooled (pair-wise) 0.0% 1.6 (0.59, 4.1)
Indirect (back-calculated) 0.62 (0.20, 2.)
Pooled (network) 24.7% 1.1 (0.53, 2.3)
Placebo vs Paroxetine
Wang XW 2009 0.050 (0.018, 0.14)
Pooled (pair-wise) 0.051 (0.010, 0.22)
Indirect (back-calculated) NA
Pooled (network) 0.049 (0.011, 0.22)
0.05
1
20
