# Supplementary material for: Treatments to post-stroke depression, which is more effective to HAMD improvement? A network meta-analysis
Source: Front Pharmacol. 2022 Dec 19;13:1035895. doi: 10.3389/fphar.2022.1035895 (PMC9806231; doi:10.3389/fphar.2022.1035895)

Figure S2 | Forest plots for the consistency of efficacy indicators. (A) HAMD change at end of 4th week. (B) HAMD change at end of 8th week. (C) Percentage of patients with 50% improvement in HAMD.

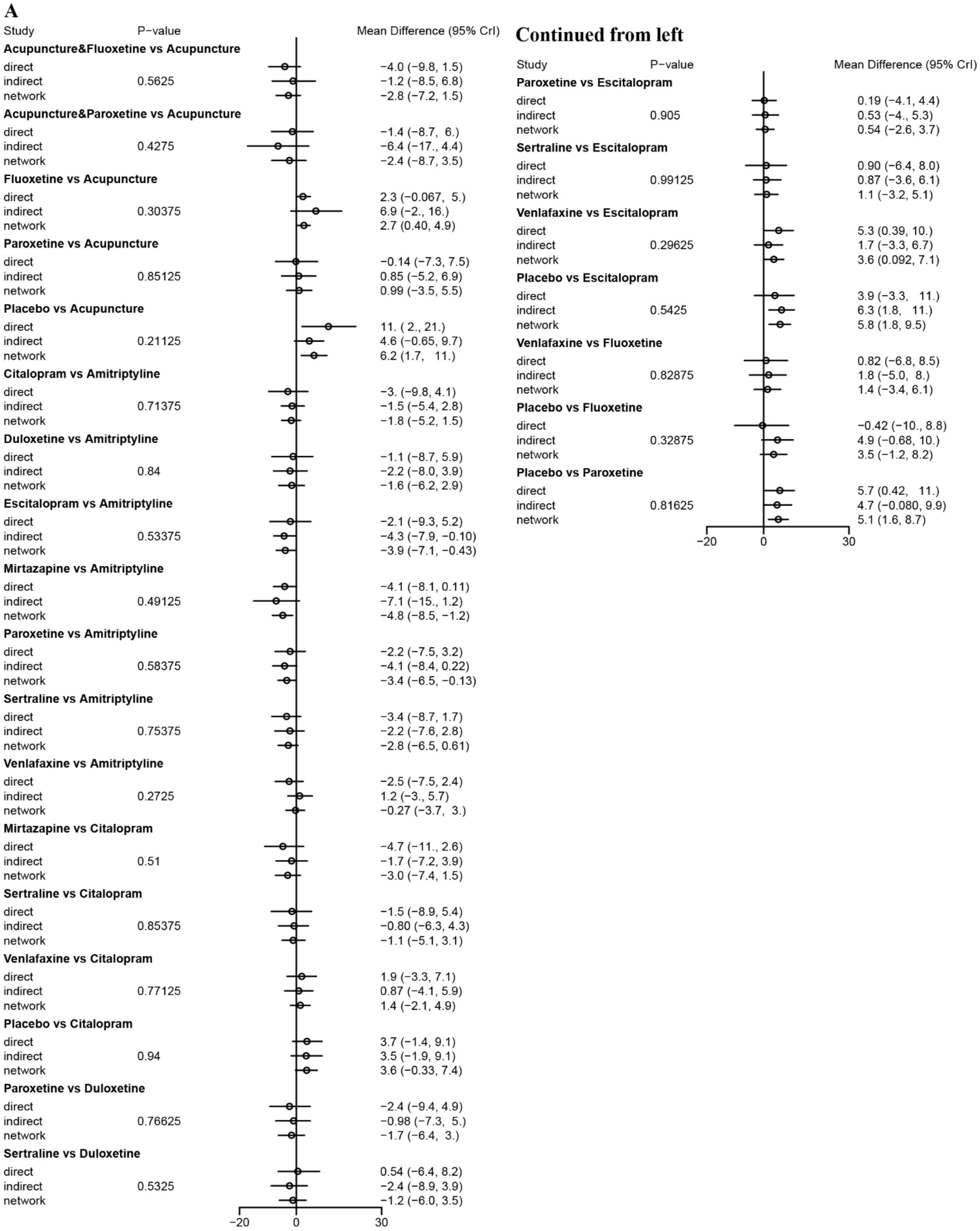

B

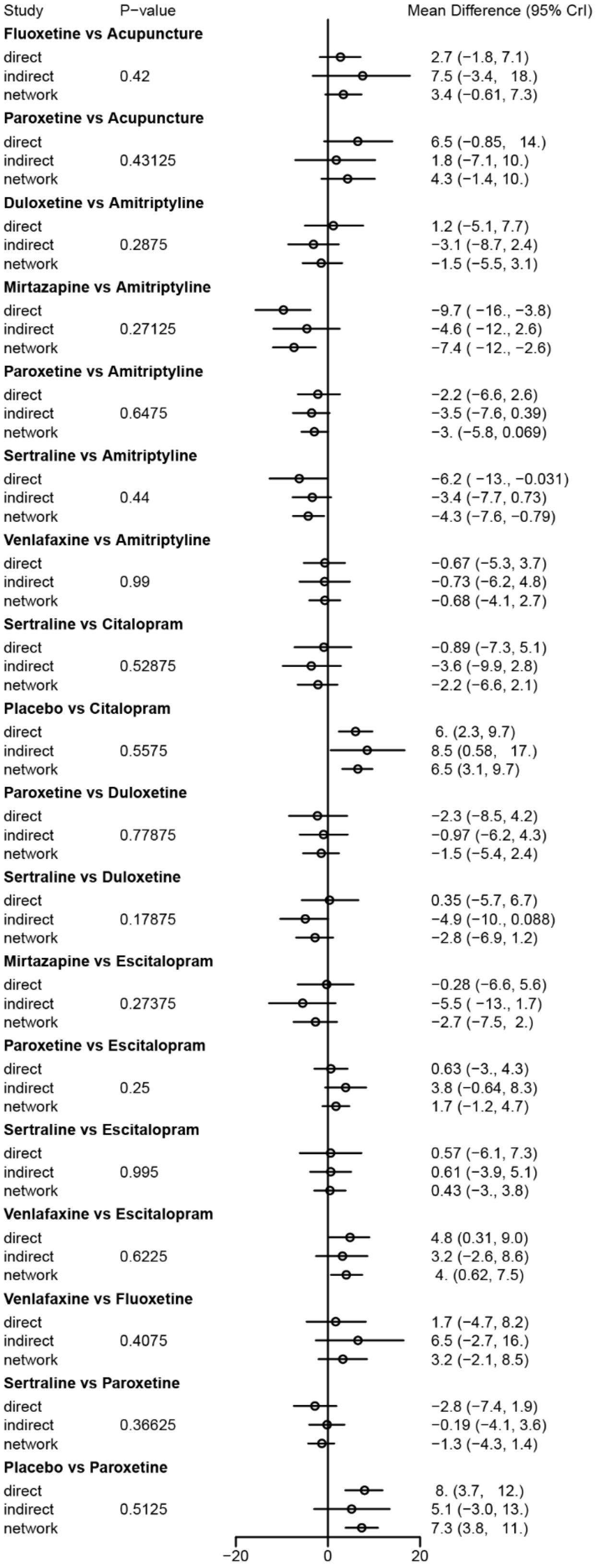

Study
P−value
Mean Difference (95% CrI)
Fluoxetine vs Acupuncture
direct 2.7 (−1.8, 7.1)
indirect 0.42 7.5 (−3.4, 18.)
network 3.4 (−0.61, 7.3)
Paroxetine vs Acupuncture
direct 6.5 (−0.85, 14.)
indirect 0.43125 1.8 (−7.1, 10.)
network 4.3 (−1.4, 10.)
Duloxetine vs Amitriptyline
direct 1.2 (−5.1, 7.7)
indirect 0.2875 −3.1 (−8.7, 2.4)
network −1.5 (−5.5, 3.1)
Mirtazapine vs Amitriptyline
direct −9.7 (−16., −3.8)
indirect 0.27125 −4.6 (−12., 2.6)
network −7.4 (−12., −2.6)
Paroxetine vs Amitriptyline
direct −2.2 (−6.6, 2.6)
indirect 0.6475 −3.5 (−7.6, 0.39)
network −3. (−5.8, 0.069)
Sertraline vs Amitriptyline
direct −6.2 (−13., −0.031)
indirect 0.44 −3.4 (−7.7, 0.73)
network −4.3 (−7.6, −0.79)
Venlafaxine vs Amitriptyline
direct −0.67 (−5.3, 3.7)
indirect 0.99 −0.73 (−6.2, 4.8)
network −0.68 (−4.1, 2.7)
Sertraline vs Citalopram
direct −0.89 (−7.3, 5.1)
indirect 0.52875 −3.6 (−9.9, 2.8)
network −2.2 (−6.6, 2.1)
Placebo vs Citalopram
direct 6. (2.3, 9.7)
indirect 0.5575 8.5 (0.58, 17.)
network 6.5 (3.1, 9.7)
Paroxetine vs Duloxetine
direct −2.3 (−8.5, 4.2)
indirect 0.77875 −0.97 (−6.2, 4.3)
network −1.5 (−5.4, 2.4)
Sertraline vs Duloxetine
direct 0.35 (−5.7, 6.7)
indirect 0.17875 −4.9 (−10., 0.088)
network −2.8 (−6.9, 1.2)
Mirtazapine vs Escitalopram
direct −0.28 (−6.6, 5.6)
indirect 0.27375 −5.5 (−13., 1.7)
network −2.7 (−7.5, 2.)
Paroxetine vs Escitalopram
direct 0.63 (−3., 4.3)
indirect 0.25 3.8 (−0.64, 8.3)
network 1.7 (−1.2, 4.7)
Sertraline vs Escitalopram
direct 0.57 (−6.1, 7.3)
indirect 0.995 0.61 (−3.9, 5.1)
network 0.43 (−3., 3.8)
Venlafaxine vs Escitalopram
direct 4.8 (0.31, 9.0)
indirect 0.6225 3.2 (−2.6, 8.6)
network 4. (0.62, 7.5)
Venlafaxine vs Fluoxetine
direct 1.7 (−4.7, 8.2)
indirect 0.4075 6.5 (−2.7, 16.)
network 3.2 (−2.1, 8.5)
Sertraline vs Paroxetine
direct −2.8 (−7.4, 1.9)
indirect 0.36625 −0.19 (−4.1, 3.6)
network −1.3 (−4.3, 1.4)
Placebo vs Paroxetine
direct 8. (3.7, 12.)
indirect 0.5125 5.1 (−3.0, 13.)
network 7.3 (3.8, 11.)
−20
0
20

C

Study	P−value	Odds Ratio (95% CrI)
**Acupuncture&Paroxetine vs Acupuncture**		
direct		1.1 (0.29, 4.)
indirect	0.5675	0.52 (0.065, 5.1)
network		0.90 (0.26, 3.2)
**Duloxetine vs Amitriptyline**		
direct		0.89 (0.18, 3.7)
indirect	0.53375	1.7 (0.43, 6.1)
network		1.2 (0.48, 3.3)
**Mirtazapine vs Amitriptyline**		
direct		2.5 (0.93, 6.8)
indirect	0.10625	19. (2.1, 1.8e+02)
network		3.6 (1.4, 9.6)
**Paroxetine vs Amitriptyline**		
direct		1.7 (0.62, 4.4)
indirect	0.45	0.96 (0.30, 3.1)
network		1.3 (0.62, 2.7)
**Sertraline vs Amitriptyline**		
direct		1.3 (0.30, 5.9)
indirect	0.89875	1.5 (0.55, 4.)
network		1.4 (0.65, 3.2)
**Venlafaxine vs Amitriptyline**		
direct		1.9 (0.47, 7.8)
indirect	0.3875	0.82 (0.17, 3.2)
network		1.3 (0.44, 3.5)
**Mirtazapine vs Citalopran**		
direct		8.4 (1.6, 55.)
indirect	0.09625	1.1 (0.19, 6.3)
network		3.1 (0.93, 10.)
**Venlafaxine vs Citalopran**		
direct		0.76 (0.27, 2.1)
indirect	0.115	5.8 (0.57, 63.)
network		1.1 (0.41, 2.7)
**Paroxetine vs Duloxetine**		
direct		1.3 (0.27, 6.3)
indirect	0.7175	0.91 (0.28, 2.9)
network		1.1 (0.40, 2.8)
**Sertraline vs Duloxetine**		
direct		0.67 (0.17, 2.7)
indirect	0.2975	1.8 (0.46, 6.1)
network		1.2 (0.42, 3.1)
**Paroxetine vs Escitalopram**		
direct		0.92 (0.35, 2.4)
indirect	0.92375	0.86 (0.21, 3.3)
network		0.92 (0.42, 1.9)
**Sertraline vs Escitalopram**		
direct		0.75 (0.15, 3.4)
indirect	0.6775	1.1 (0.38, 3.1)
network		0.99 (0.41, 2.5)
**Venlafaxine vs Escitalopram**		
direct		1.0 (0.26, 4.3)
indirect	0.7275	0.71 (0.12, 3.8)
network		0.87 (0.31, 2.4)
**Sertraline vs Paroxetine**		
direct		1.6 (0.61, 4.0)
indirect	0.23875	0.68 (0.23, 2.0)
network		1.1 (0.51, 2.3)

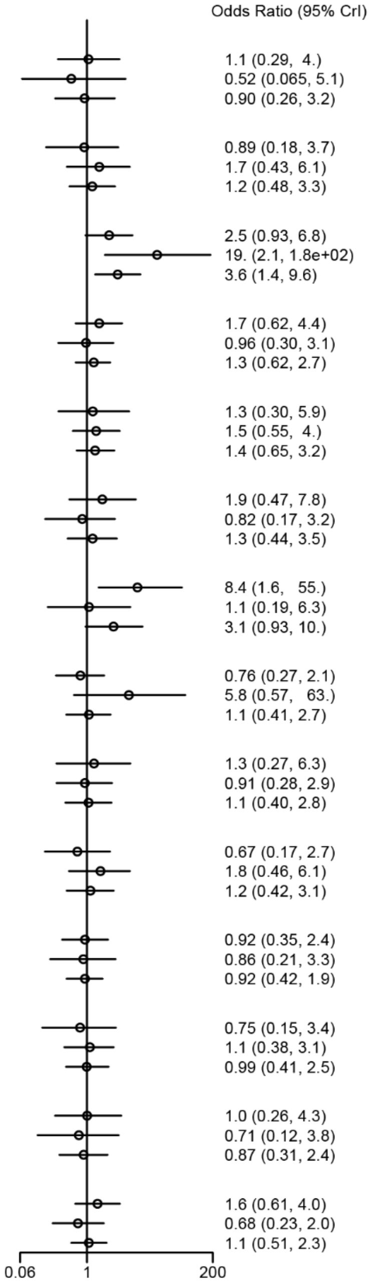

Supplement: Supplementary file 1 [file DataSheet1.zip › SupplementaryMaterialFigureS2.pdf]
