# Supplementary material for: Treatments to post-stroke depression, which is more effective to HAMD improvement? A network meta-analysis
Source: Front Pharmacol. 2022 Dec 19;13:1035895. doi: 10.3389/fphar.2022.1035895 (PMC9806231; doi:10.3389/fphar.2022.1035895)
Supplement: Supplementary file 1 [file DataSheet1.zip › SupplementaryMaterialTable1.docx]

**Supplement Materials**

**Table 1: Characteristics of the Included Studies and Outcome Events**

| **Study** | **Countries** | **Publications** | **Treatment group,**  **(No. of participants)** | **Baseline of HAMD (Mean±SD)** | **Female (%)** | **Mean age** **±SD** **(year)** | **Outcome Events** |
| --- | --- | --- | --- | --- | --- | --- | --- |
| Stefan Fruehwald 2003 | Austria | J Neurol | FLU (26)  vs. PLA (24) | FLU 32.8±12.7  PLA 30.3±15.0 | FLU 53.8%  PLA 29.1% | FLU 64.8±13.8  PLA 64.0±14.3 | a |
| Dong ZY 2003 | China | Chinese Joumal of Clinical Rehabilitation | ACU (42)  vs. FLU (38) | ACU 27.3±5.3  FLU 27.7±7.4 | ACU 42.5%  FLU 45.6% | N/A | a |
| Zhang CZ 2005 | China | Journal of Traditional Chinese Medicine | ACU (45)  vs. FLU (45) | ACU 23.78±4.57  FLU 25.36±3.58 | ACU 44.4%  FLU 40% | ACU 55.5±6.27  FLU 58.1±8.91 | a,c |
| Zhao FT 2005 | China | Chinese Journal of Clinical Rehabilitati | CIT (42)  vs.VEN (40) | CIT 32.3±8.4  VEN 32.4±9.3 | CIT 42.8%  VEN 47.5% | CIT 58.16±8.49  VEN 61.45±8.24 | a,c |
| Seo Young Gu 2006 | Korea | Brain Stimulation | rTMS (12)  vs. PLA (12) | rTMS 10±1.3  PLA 10±0.9 | rTMS 50%  PLA 71.42% | rTMS 58.1±8.7  PLA 58.3±7.8 | a |
| Zhao YS 2006 | China | journal of clinical acupuncture and moxibustion | ACU (23)  vs. FLU (21) | ACU 24.8±1.3  FLU 25.2±1.5 | ACU 47.8%  FLU 52.3% | N/A | a,c |
| Luo RQ 2006 | China | Chin J Rehabil Theory Practice | PAR (42)  vs.AMI (40) | PAR 28.8±6.7  AMI 29.2±5.1 | PAR 26.1%  AMI 30% | PAR 61.2±4.5  AMI 60.5±5.3 | a,b,c |
| Dong JP 2007 | China | Chinese Acupuncture | ACU (36)  vs. FLU (34) | ACU 23.98±3.69  FLU 24.12±3.17 | ACU 36.1%  FLU 44.1% | ACU 59.21±7.56  FLU 56.61±8.21 | a,c |
| Li ZJ 2007 | China | Modern Journal of Integrated Traditional Chinese and Western Medicine | PAR (44)  vs.PLA (42) | PAR 23.9±5.78  PLA 23.7±7.54 | PAR 36.3%  PLA 38.1% | PAR 59.2±12  PLA 58.9±12.1 | a,b |
| Luca Cravello 2009 | Italy | human psychopharmacology | VEN (25)  vs. FLU (25) | VEN 17±4.5  FLU 19.2±4.4 | VEN 56%  FLU 64% | VEN 64.2±14.1  FLU 65.9±12.7 | a,b |
| Zhou XH 2009 | China | Shandong Medical Journal | HM (45)  vs. FLU (45) | HM 23.15±4.11  FLU 24.02±4.38 | HM 51.1%  FLU 46.6% | N/A | a,b |
| Wu JF 2009 | China | Chinese Journal of Practical Nervous Disease | SER (42)  vs.AMI (42) | SER 25.4±3.1  AMI 24.8±4.6 | 40.4% | 63.2±6.5 | a,c |
| Chen HC 2009 | China | Guiding Journal of Traditional Chinese Medicine and Pharmacy | HM (40)  vs. FLU (40) | HM 24.24±6.27  FLU 27.35±5.68 | HM 40%  FLU 45% | HM 63.4±10.6  FLU 72.0±6.8 | a,c |
| Wang XW 2009 | China | Chinese Community Doctors | PAR (55)  vs.PLA (55) | PAR 23.3±6.7  PLA 22.8±7.8 | 31.8% | 56±15 | a,b,c |
| Wu JP 2010 | China | Acupuncture Research | ACU (150)  vs.FLU (150) | ACU 29.4±5.6  FLU 28.7±6 | ACU 52.6%  FLU 54% | ACU 56.2±9.2  FLU 55.7±9.4 | b,c |
| Nie RR 2011 | China | Chinese Acupuncture | ACU (33)  vs.FLU (30) | ACU 28.37±4.34  FLU 27.48±4.67 | ACU 48.57%  FLU 42.85% | ACU 64.2±9.85  FLU 63.1±9.55 | a,c |
| Huang PZ 2011 | China | Health industry in China | HM (30)  vs. FLU (30) | HM 19.6±4.2  FLU 19.3±4.4 | HM 63.3%  FLU 66.6% | N/A | a,c |
| Li J 2011 | China | Chinese Journal of Practical Nervous Disease | DUL (45)  vs.AMI (45) | DUL 29.55±2.78  AMI 29.8±3.26 | DUL 33.3%  AMI 35.5% | DUL 59.5±3  AMI 59.6±4.8 | a,b,c |
| Wang L 2011 | China | Chinese Journal of Medicinal Guide | MIR (42)  vs.AMI (42) | MIR 25.4±3.1  AMI 24.8±4.6 | 40.4% | 63.2±6.5 | a,c |
| Li HJ 2011 | China | Chinese Acupuncture | ACU (23)  vs. FLU (20) | ACU 24.8±5  FLU 26.7±4.6 | ACU 47.8%  FLU 50% | ACU 56.7±14.4  FLU 59.4±14.1 | a |
| Shao J 2012 | China | Chinese Journal of Hospital Pharmacy | AMI (66)  vs.ESC (66) | AMI 22.0±4.7  ESC 22.5±4.9 | N/A | N/A | a |
| Chen WB 2012 | China | Zhejiang Praetical MMicine | DUL (48)  vs.SER (48) | DUL 23.21±5.15  SER 23.56±4.37 | DUL37.5%  SER 41.6% | DUL 63±5.56  SER 65±4.78 | a,b,c |
| Li CX 2012 | China | Guide of China Medicine | CIT (53)  vs.SER (53) | CIT 29.59±1.76  SER 29.76±1.65 | N/A | N/A | a,b |
| Liu J 2012 | China | China Modern Doctor | CIT (40)  vs.VEN (40) | CIT 33.6±4.2  VEN 32.3±4.6 | CIT 47.5%  VEN 42.5% | CIT 62.7±8.7  VEN 61.3±7.7 | a,c |
| Wu SG 2013 | China | Hei long jiang medical journal | ESC (40)  vs.PAR (40) | ESC 25.48±2.7  PAR 25.28±2.75 | ESC 40.0%  PAR 47.5% | ESC 67.5±9.3  PAR 68.2±11.2 | a,b,c |
| Nie RR 2013 | China | Chinese Acupuncture | ACU (42)  vs. PAR (40)  vs. ACU+PAR (41) | ACU 30.37±4.24  PAR 31.46±4.17  ACU+PAR 31.35±4.07 | ACU 54.7%  PAR 55%  ACU+PAR 58.5% | ACU 64±10  PAR 64±10  ACU+PAR 63±10 | a,c |
| Huang C 2014 | China | Journal of Practical Cardio-cerebral pulmonary vascular diseases | VEN (100)  vs.ESC (100) | VEN 30.76±4.19  ESC 29.61±4.25 | VEN 42%  ESC 40% | VEN 69.2±2.5  ESC 69.2±2.1 | a,b |
| Li ZD 2014 | China | Chinese Journal of Contemporary Medicine | VEN (45)  vs. AMI (46) | VEN 28.8±6.2  AMI 29.1±6.3 | N/A | N/A | a,b,c |
| Li XF 2014 | China | Chin J Mod Drug Appl | PAR (40)  vs.ESC (40) | PAR 27.06±4.09  ESC 27.22±3.99 | PAR 57.5%  ESC 62.5% | PAR 38.64±5.93  ESC 38.91±5.73 | a,b,c |
| Wei HL 2014 | China | Chin J of Clinical Rational Drug Use | CIT (40)  vs.AMI (40) | CIT 22.7±2.6  AMI 22.3±2.8 | CIT 32.5  AMI 37.5% | CIT 70.1±6.9  AMI 69.2±7.3 | a |
| Li XZ 2015 | China | Frontier Journal of Medicine | MIR (42)  vs.AMI (42) | MIR 30.11±3.92  AMI 41.41±5.22 | MIR 42%  AMI 42% | MIR 58±2.6  AMI 56±3.8 | a,b |
| Dong Y 2015 | China | J Phys Ther Sci | HBO (30)  vs. FLU (30)  vs. HBO+FLU (30) | HBO 20.1±5.7  FLU 19.8±4.5  HBO+FLU 22.8±3.3 | HBO 46.6%  FLU 43.3%  HBO+FLU 40% | HBO 63±8.1  FLU 65±7.9  HBO+FLU 66±5.9 | a,c |
| Wang GQ 2015 | China | Anhui Medical Journal | SER (75)  vs.AMI (75) | SER 31.57±3.42  AMI 32.14±4.63 | SER 48%  AMI 45.3% | SER 65.2±6.7  AMI 65.8±6.1 | a |
| Li HX 2015 | China | Joumal of Qiqihar University of Medicine | VEN (74)  vs.AMI (74) | VEN 27.6±5.4  AMI 26.3±5.7 | 45.9% | 52.6±2.5 | a,b,c |
| Chen ZY 2015 | China | Journal of International Psychiatry | CIT (48)  vs. PLA (48) | CIT 26.6±6.7  PLA 27.4±7 | CIT 47.9%  PLA 45.8% | CIT 60.8±9.6  PLA 61.0±9.5 | b |
| Feng QL 2015 | China | J Clin Psychosom Dis | ESC (40)  vs.SER (40) | ESC 25.47±2.71  SER 25.29±2.76 | ESC 42.45%  SER 47.5% | ESC 65±4.5  SER 65.3±5.1 | a,b,c |
| Sun YT 2015 | China | Chinese Acupuncture | ACU (31)  vs. FLU (31)  vs. ACU+FLU (31) | ACU 23.78±5.46  FLU 24.23±6.16  ACU+FLU 23.62±6.23 | ACU 51.6%  FLU 51.6%  ACU+FLU 54.8% | ACU 67±4  FLU 69±5  ACU+FLU 68±5 | a,c |
| Sun PY 2015 | China | Chinese Acupuncture | PAR (30)  vs. ACU+PAR (33) | PAR 29.34±10.22  ACU+PAR 29.25±9.73 | PAR 50%  ACU+PAR 36.4% | PAR 58±8  ACU+PAR 59±7 | a,c |
| Song WZ 2016 | China | Chinese Journal of Trauma and Disability Medicine | MIR (46)  vs.CIT (45) | MIR 23.73±2.07  CIT 22.94±1.98 | MIR 45.6%  CIT 48.8% | MIR 63.2±5.1  CIT 62.9±5.7 | a,c |
| Li R 2016 | China | China Journal of Pharmaceutical Economics | PAR (103)  vs.AMI (103) | PAR 28±5  AMI 27±4 | PAR 40.7%  AMI 36.8% | PAR 68.5±2.1  AMI 66.54±1.7 | a,b,c |
| Hu JQ 2016 | China | Chinese Journal of Integrative Medicine on Cardio | MIR (40)  vs.AMI (40) | MIR 20.96±4.52  AMI 20.47±3.65 | MIR 40%  AMI 37.5% | MIR 61.1±5.6  AMI 60.7±6.1 | a |
| Wu SW 2016 | China | Chinese Journal of Practical Nervous Disease | VEN (44)  vs.ESC (44) | VEN 29.8±3.6  ESC 30.6±4.4 | VEN 45.4%  ESC 47.5% | VEN 72.8±10.1  ESC 74.6±10.4 | a,b,c |
| Feng YH 2016 | China | Chinese Journal of Practical Nervous Disease | PAR (57)  vs. SER (58) | PAR 23.6±2.5  SER 23.5±2.5 | PAR 45.6%  SER 44.8% | PAR 60.7±6.3  SER 60.6±6.6 | b,c |
| Li PW 2017 | China | China Pharmacy | SER (42)  vs. PAR (49) | SER 24.75±6.03  PAR 24.46±5.89 | SER 42.8%  PAR 28.5% | SER 68.4±9.1  PAR 66.7±8.5 | b,c |
| Hu J 2017 | China | Capital Journal of Food and Medicine | ESC (50)  vs. MIR (50) | ESC 30.17±6.06  MIR 31.36±5.27 | ESC 40%  MIR 44% | ESC 56.42±5.18  MIR 54.21±6.32 | b |
| Li MH 2017 | China | Journal of Traditional Chinese Medicine | ACU (29)  vs. FLU (29) | ACU 19±7  FLU 20±8 | ACU 44.8%  FLU 41.3% | ACU69±7  FLU 68±7 | a,b |
| Li J 2017 | China | Journal of Hebei Medical University | ESC (64)  vs.PLA (62) | ESC 21.83±1.46  PLA 21.62±1.41 | ESC 34.3%  PLAV35.4% | ESC 62.02±9.8  PLA 62.35±9.31 | a |
| Wang G 2017 | China | Practical geriatrics | ESC (40)  vs.PAR (40) | ESC 29.55±4.21  PAR 29.82±4.53 | ESC 42.5%  PAR 45% | ESC 67.58±6.08  PAR 67.15±5.95 | a,b,c |
| Lin Z 2017 | China | Chinese Acupuncture & Moxibustion | ACU (30)  vs.PAR (30) | ACU 26.72±7.63  PAR 25.97±8.16 | ACU 56.6%  PAR 53.3% | ACU 59±9  PAR 58±8 | b,c |
| Ye BJ 2018 | China | China Modern Doctor | SER (46)  vs. AMI (46) | SER 27.98±3.31  AMI 28.18±4.08 | SER 47.8%  AMI 43.4% | SER 70.08±6.41  AMI 69.81±6.45 | b |
| Chen AW 2018 | China | Chinese Acupuncture | PLA (30)  vs. ACU (30) | PLA 36.6±9.5  BEL 35.7±10 | PLA 56%  ACU 53.3% | PLA 58±11  ACU 57±11 | a |
| Bai W 2018 | China | Guizhou Medical Journal | PAR (75)  vs.DUL (75) | PAR 23.92±1.71  DUL 23.96±1.73 | PAR 45.3%  DUL 48% | PAR 58.12±2.37  DUL 59.13±2.35 | a,b,c |
| Chen M 2018 | China | Medicine | PAR (35)  vs.PLA (35) | PAR 27.9±6.8  PLA 26.0±5.9 | PAR 34.3%  PLA 42.9% | PAR 62.5±11.4  PLA 64.1±12.3 | b |
| Yang HM 2019 | China | Yunnan Medical Journal | rTMS+PAR (49)  vs. PAR (49) | rTMS+PAR 21.47±1.56  PAR 22.25±1.49 | rTMS+PAR 42.8%  PAR 40.8% | rTMS+PAR 58.42±7.16  PAR 58.72±6.94 | b,c |
| Cao JX 2020 | China | Restorative Neurology and Neuroscience | CIT (52)  vs. PLA (47) | CIT 12.23±3.12  PLA 11.98±3.41 | N/A | N/A | a,b |
| Liu FJ 2020 | China | Journal of reflexology and Rehabilitation Medicine | rTMS+PAR (34)  vs. PAR (34) | rTMS+PAR 25.22±1.25  PAR 26.09±0.29 | rTMS+PAR 44.12%  PAR 35.29% | rTMS+PAR 54.92±11.8  PAR 55.2±12.6 | b,c |
| Hu M 2020 | China | Journal of Chinese Contemporary Medicine | rTMS+PAR (40)  vs. PAR (40) | rTMS+PAR 28.5±5.1  PAR 28.1±5.3 | rTMS+PAR 37.5%  PAR 42.5% | rTMS+PAR 48.7±5.4  PAR 49.3±5.8 | b |
| Wang RT 2020 | China | Neural Injury And Functional Reconstruction | rTMS+PAR (76)  vs. PAR (76) | rTMS+PAR 28.34±5.13  PAR 27.25±5.01 | rTMS+PAR 59.2%  PAR 56.5% | rTMS+PAR 68.52±5.71  PAR 68.39±5.02 | b,c |
| You YL 2020 | China | Journal of Traditional Chinese Medicine | ACU+FLU (35)  vs. FLU (35) | N/A | 38% | N/A | a |
| Huang WL 2021 | China | World Journal of Integrated Traditional and Western Medicine | ACU (30)  vs. FLU (30) | ACU 11.89±2.73  FLU 12.07±3.08 | ACU 50%  FLU 43.3% | ACU 63.51±6.09  FLU 64.14±5.47 | a,c |

PLA: Placebo; ACU: Acupuncture; FLU: Fluoxetine; PAR: Paroxetine; CIT: Citalopram; rTMS: repetitive transcranial magnetic stimulation; HBO: Hyperbaric oxygen; VEN: Venlafaxine; HM: Herb medicine; AMI: Amitriptyline; ESC: Escitalopram; SER: Sertraline; DUL: Duloxetine; MIR: Mirtazapine; N/A: not application.

(a) HAMD change at end of 4th week, (b) HAMD change at end of 8th week, (c) Percentage of patients with 50% improvement in HAMD.
